# Tripeptides as Integrin-Linked Kinase Modulating Agents Based on a Protein–Protein Interaction with α-Parvin

**DOI:** 10.1021/acsmedchemlett.1c00183

**Published:** 2021-07-15

**Authors:** Javier Garcia-Marin, Mercedes Griera-Merino, Alejandra Matamoros-Recio, Sergio de Frutos, Manuel Rodríguez-Puyol, Ramón Alajarín, Juan J. Vaquero, Diego Rodríguez-Puyol

**Affiliations:** † Departamento de Química Orgánica y Química Inorgánica, Universidad de Alcalá, Alcalá de Henares 28805, Spain; # Fundación de Investigación Biomédica, Unidad de Nefrología del Hospital Príncipe de Asturias y Departamento de Medicina y Especialidades Médicas, Universidad de Alcalá, Alcalá de Henares 28805, Spain; ⊥ Departamento de Biología de Sistemas, Universidad de Alcalá, Alcalá de Henares 28805, Spain; ‡ Instituto Ramón y Cajal de Investigación Sanitaria (IRYCIS), Ctra. Colmenar Viejo, km. 9100, Madrid 28034, Spain; § Instituto de Investigación Química Andrés Manuel del Río (IQAR), Universidad de Alcalá, Alcalá de Henares 28805, Spain; ○ Fundación Renal Iñigo Álvarez de Toledo (FRIAT) y Instituto de Salud Carlos III (REDinREN), Madrid 28029, Spain; || Graphenano Medical Care, S.L, Yecla 30510, Spain

**Keywords:** integrin-linked kinase, ILK, parvin, tripeptide, chronic kidney disease, protein−protein interaction, hot spot

## Abstract

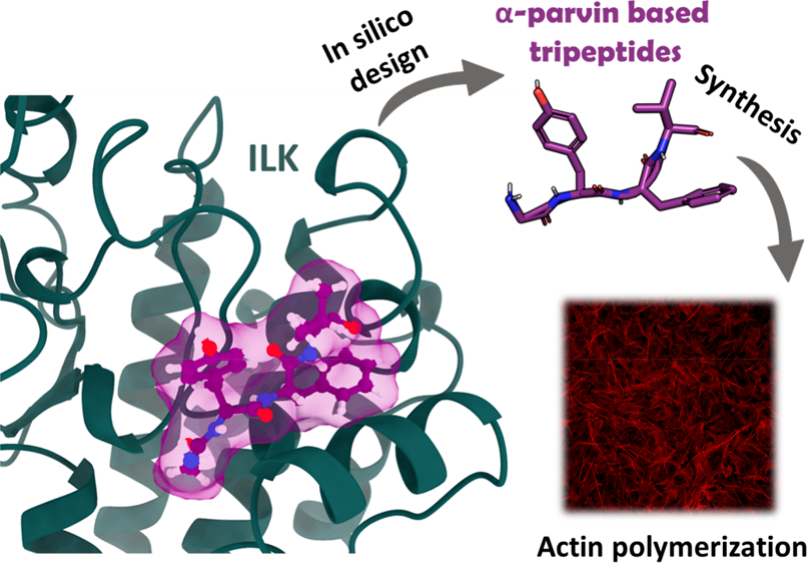

Integrin-linked kinase (ILK) has emerged as a controversial pseudokinase protein that plays a crucial role in the signaling process initiated by integrin-mediated signaling. However, ILK also exhibits a scaffolding protein function inside cells, controlling cytoskeletal dynamics, and has been related to non-neoplastic diseases such as chronic kidney disease (CKD). Although this protein always acts as a heterotrimeric complex bound to PINCH and parvin adaptor proteins, the role of parvin proteins is currently not well understood. Using in silico approaches for the design, we have generated and prepared a set of new tripeptides mimicking an α-parvin segment. These derivatives exhibit activity in phenotypic assays in an ILK-dependent manner without altering kinase activity, thus allowing the generation of new chemical probes and drug candidates with interesting ILK-modulating activities.

The control of cytoskeletal architecture is one of the most crucial events for cell shape, growth, survival, and differentiation.^1^ A plethora of different components are involved in this process, including biomacromolecules, which play their own role. Of these, integrin-linked kinase (ILK) has become one of the most important and fascinating such molecules. Discovered in 1996 by Dedhar and co-workers as a integrin-β1 subunit binding protein,^2^ ILK has been known to be a kinase for a long time.^2−5^ However, as its crystal structure revealed a pseudoactive kinase catalytic site (PDB ID: 3KMW), this putative activity as a protein kinase has been questioned and become quite controversial.^6−9^ Nonetheless, studies with this molecule have led to its validation as a promising therapeutic target for cancer.^3,10,11^

Several studies have demonstrated that ILK acts as a tight heterotrimeric complex in vivo with the two adaptor proteins PINCH and parvin.^7,9,12^ The presence of this ternary complex in focal adhesions is critical for the outside-in signaling initiated by integrin activation. As a result of its N-terminal ankyrin repeating domain, ILK is able to interact with PINCH,^13,14^ whereas the C-terminal kinase-like domain recognizes the C-terminal calponin homology domain (CH2) of α-parvin.^15,16^

Studies carried out by Rodriguez-Puyol et al. showed that ILK activation and inhibition are tightly linked to the development of chronic kidney disease (CKD).^17,18^ In addition, α-parvin may play a critical role in the kidney development and function;^19^ thus, given its interaction with ILK, this protein may represent a potential target for the development of new drugs against CKD. Furthermore, as parvin proteins anchor ILK directly to the actin bundles,^20^ their modulation may have interesting effects from a cellular point of view.

Recently, our group has started a new research area aimed at modulating ILK as a therapeutic target for CKD. In this context, we propose to explore the potential of this target in CKD by designing new molecules that are able to modulate ILK. As protein–protein interactions have emerged as a very promising source of druggable targets,^21,22^ we decided to start from scratch, basing our approach on a study of the ILK−α-parvin interaction. This was motivated by the existence of several crystal structures published in the PDB,^6^ prior to this study (3KMW and 3KMU) between the ILK kinase domain and α-parvin CH2 domain, which show a well-defined topology between the two globular domains of both partners.

As the crystal structures of the ILK domain (hereinafter ILK) and α-parvin CH2 have been solved by X-ray crystallography, PDB: 3KMW (2.0 Å) was selected for computational studies. Initially, the FT-Map algorithm,^23^ which is based on the docking of different organic probes, was employed because of its success in identifying experimentally validated hot spots.^24,25^ Up to nine different hot spots were identified on the ILK surface using this tool (Figure S1 A). However, for analysis, we chose the most densely populated clusters near the α-parvin interface. The first hot spot in the FT-Map ranking corresponded to the ATP binding pocket, thus validating this computational approach for identifying important regions for small molecule binding. A second one, placed in the back region of the ATP cavity was discarded due to the lack of experimental evidence for an ILK-parvin interaction at this position. On the other hand, a third hot spot was located at the position where α-parvin interacts with ILK via an uncoiled loop that connects the αD and αC CH2 helixes (Figure 1A, B). Interestingly, when the chemical probes of this hot spot cluster were examined in greater depth, we observed that they were concentrated in a small swallow pocket that accommodates α-parvin Phe307 (Figure 1B). A careful inspection of the 3KMW PDB revealed that, in the crystal structure, Phe307 establishes two hydrogen bonds via the carbonyl and NH groups with the Met350 backbone in ILK, whereas the phenyl ring contributes to this binding via van der Waals interactions within the pocket. No relevant interactions were found in the adjacent residues Tyr306 and Val308, except for Gly305, which is hydrogen-bonded ILK Asn397 via its carbonyl group to (Figure 1C). To carry out a consensus approach and support this initial analysis, we also selected the HotPoint method for hot spot identification. This tool is able to predict hot spots using an empirical model based on the occlusion from solvent and a knowledge-based pair potential of residues present at the interaction interface.^26^ The server identifies contacting residues and classifies them into hot spots or not. This analysis highlighted Phe307 and Val308 as important and putative hot spots (see Figure S1B) of α-parvin in the same region as the FT-Map. Giving all these results, we hypothesized that a small peptide fragment mimicking the hot spot could modulate ILK. As such, a tripeptide with the sequence H-Tyr-Phe-Val-OH (**1**) was chosen as starting point for our design. We decided to acetylate the -NH_2_ terminal group with two different aims: to increase its chemical stability and to emulate the hydrogen bond established by Gly305 in the crystal structure. In addition, as the carboxylate group of Val308 residue fits into the hydrophobic cleft, we decided to methylate this position in our design proposal to avoid undesired electrostatic interactions for the hydrophobic environment of the pocket. Additionally, this derivatization with a methyl ester at the *C*-terminal peptide may increase its permeability through biological membranes.^27^ Prior to synthesis, conventional molecular dynamics studies were carried out to assess the behavior of both designs. Complexes between the ILK kinase domain, **1** and **2** (Ac-Tyr-Phe-Val-OMe), were simulated for 50 ns and the RMSD of the heavy atoms in the protein and peptides were measured along the simulation time (see the Supporting Information for more details). The RMSD plot for both simulations showed lower mean values for **2** than for **1** (for the peptide as well as ILK-peptide complexes), thus suggesting a higher stability for **2**. In general, these simulations proved that the initial binding mode and interactions were maintained during the simulation, especially for derivative **2**. Thus, these data suggested a better scenario for methylated peptide. As a proof of concept, we decided to prepare both compounds, **1** and **2**, to determine their activity and evaluate their potential interest.

**Figure 1:**
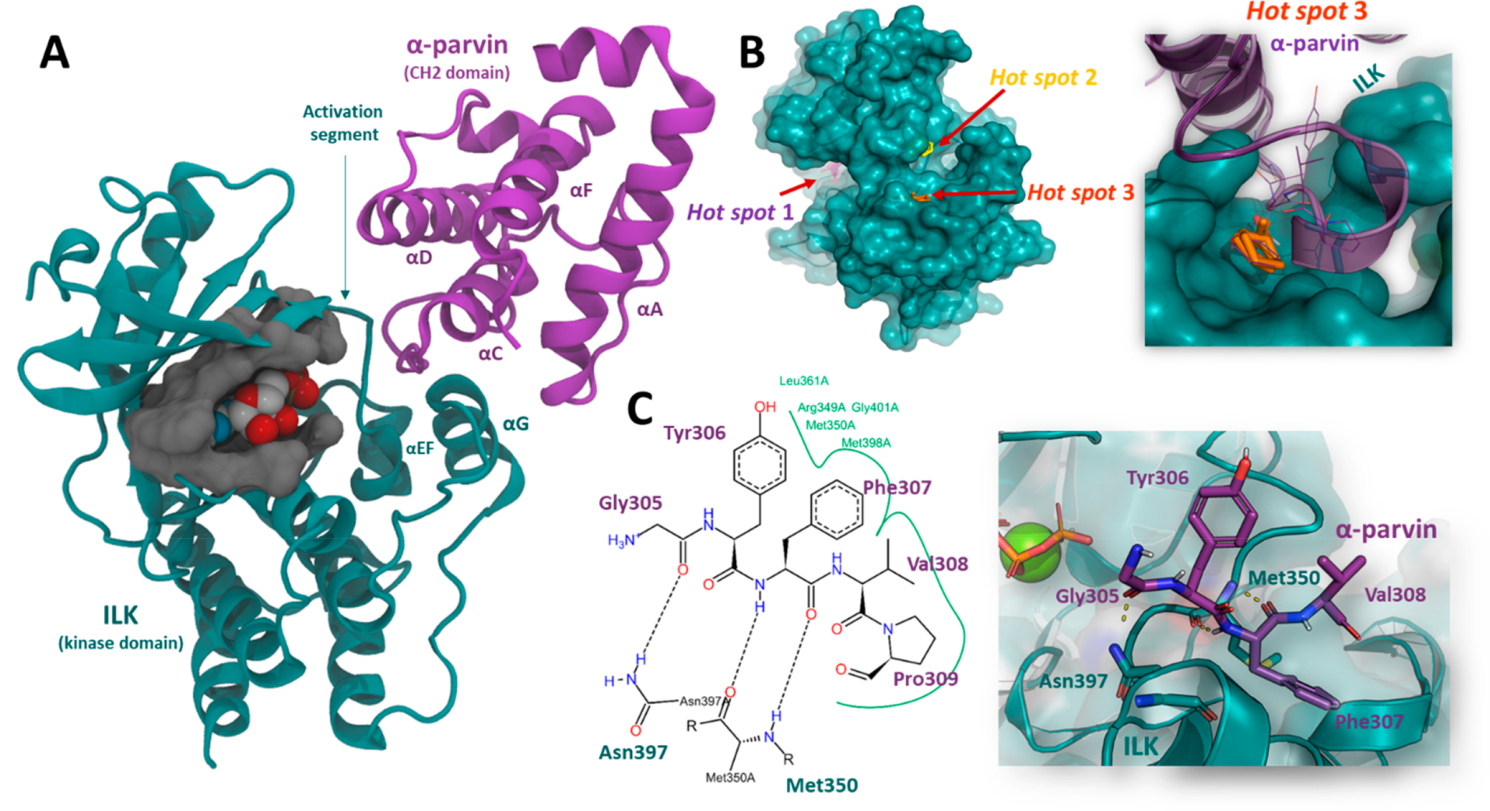
(A) Tridimensional structure of the ILK kinase domain complexed with ATP and magnesium bound to the CH2 domain of α-parvin (PDB: 3KMW). (B) Representation of the best ranked hot spot predicted by FTMap on the ILK kinase domain surface. (C) Diagram of interaction between ILK and the α-parvin peptide chain comprising residues Gly305 to Pro309 generated with *PoseView* (https://proteins.plus/) and 3D representation generated from the PDB.

Peptides **1** and **2** were prepared using standard solid-phase peptide synthesis procedures, using 2-chlorotrityl chloride resin. After applying the standard conditions for resin acid cleavage, **1** was obtained easily with high purity (see Table 1), as expected. To prepare the methyl ester derivative **2**, the cleavage-esterification procedure described by Turner was selected,^28^ and adapted to avoid the low swelling of the resin in the methanol cocktail. To overcome this issue, we changed methanol for 4 M HCl in dioxane, because of its better swelling index and commercial availability.^29^ After finding appropriate conditions, methyl ester **2** was easily obtained.

**Table 1:** Peptide Derivatives **1**–**8** (Ac-aa1-aa2-aa3-OR) and Purity at a Wavelength of 214 nm after HPLC Purification

peptide	aa1-aa2-aa3	R	purity (%)
**1**	Tyr-Phe-Val	H	>99
**2**	Tyr-Phe-Val	CH_3_	97
**3**	Ala-Phe-Val	CH_3_	>99
**4**	Tyr-Ala-Val	CH_3_	>99
**5**	Tyr-Phe-Ala	CH_3_	>99
**6**	Tyr-Phe-Ser	CH_3_	>99
**7**	Tyr-(2-NaI)-Val	CH_3_	>99
**8**	Tyr-(2-NaI)-Ser	CH_3_	94

Although several studies have supported the hypothetical kinase activity of ILK, its true nature remains somewhat controversial.^7,8^ A few such studies have used inhibitors, usually related to neoplastic diseases, to prove its mechanism of action by measuring the phosphorylation levels of both GSK-3β (Ser9) and Akt (Ser473),^30,31^ two downstream signaling substrates, as indirect ILK inhibitors. However, it appears to be widely accepted that ILK also exhibits an assembly function in the integrin signaling axis by connecting integrins to the cytoskeleton via focal adhesions.^9,32^ Despite the importance of this complementary function inside the cell, little attention has been paid to this aspect in the literature to date. Indeed, this represents an opportunity to explore the potential of ILK as a drug target in noncancer related diseases.^33^ In this context, we decided to assess both activities (kinase and assembly) for our compounds in phenotypic assays. Our first experiments were aimed to identify possible changes in the ILK kinase activity, by measuring GSK-3β and Akt phosphorylation levels. No significant changes were observed at 50 μM when the phosphorylated forms of these proteins were measured by Western blot (see Figure S3). As these results did not support a putative ILK kinase activity modulation, we analyzed the effect of our compounds on the assembly function. Several studies have shown that ILK is essential for actin cytoskeletal organization, triggering F-actin polymerization and bundling. To evaluate the assembly function, we performed a relatively simple polymerization assay to measure F-actin levels using confocal microscopy. Compound **1** did not induce any effect, whereas an increase in F-actin polymerization levels was observed after 24 h of treatment for ester **2** (Figures 2 and 3).

**Figure 2:**
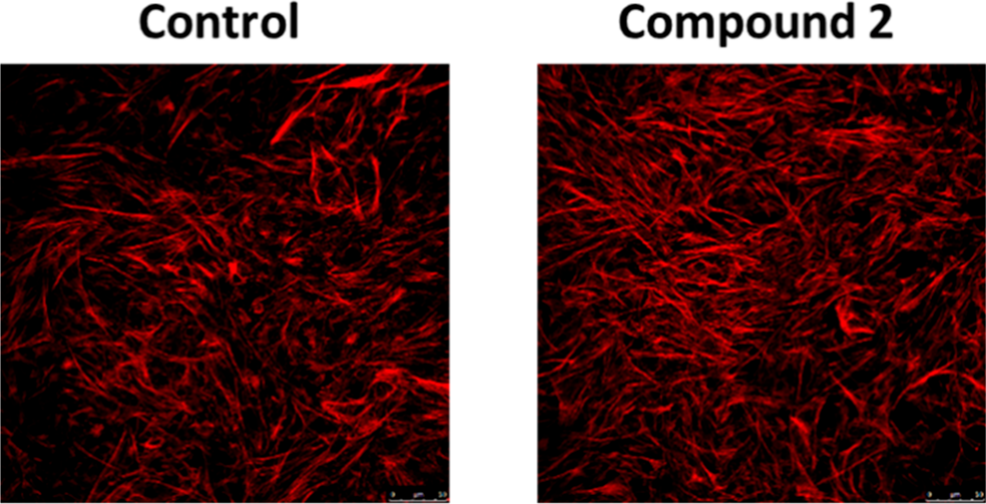
Qualitative evaluation of actin polymerization (F-actin). Human mesangial cells (HMC) were incubated in control conditions (buffer, 24 h) or with compound **2** (50 μM, 24 h), stained with Alexa 568 phalloidin and examined by confocal microscopy.

**Figure 3:**
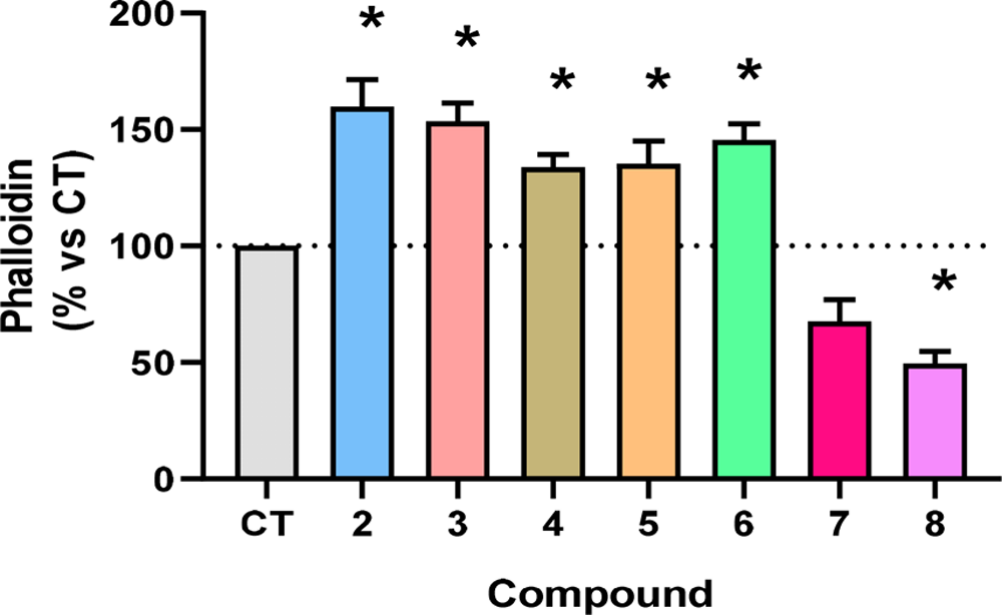
Quantitative evaluation of actin polymerization (F-actin). Human mesangial cells were incubated in control conditions (buffer, 24 h) or with compounds **2** to **8** (50 μM, 24 h), stained with Alexa 568 phalloidin and examined by confocal microscopy. The amount of F-actin was measured in three independent assays. Results are the mean ± SEM, expressed as percent of control values. *p < 0.05 vs C.

Motivated by these results, we prepared a small set of *N*-acetyl tripeptide methyl esters **3**–**8** (Table 1). After setting up our cleavage-esterification procedure, compounds were obtained with crude yields and purities ranging from moderate to high. It is noteworthy that this procedure is very sensitive to the presence of water in the dioxane solution, which leads to a decrease in purity. For those derivatives bearing a serine residue, like **6** and **8**, the nonesterified peptides were obtained together with their methyl ethers as byproducts, thereby impairing purification. The resulting peptides were purified by preparative HPLC to obtain higher purities suitable for cellular studies (>90%). These derivatives included a positional alanine scanning and introduction of a hydrophobic aromatic group in position 2 (2-NaI) of the tripeptides to fill the hot spot cavity. Moreover, a serine amino acid was introduced at the *C*-terminal position to improve solubility and increase polar interactions between the side chain and the ILK backbone.

As it occurred with compound 2, any of these newly synthesized compounds did not induce changes in the kinase activity of ILK (data not shown). However, phenotypic effects on F-actin polymerization were observed in some cases (Figure 3).

The peptides can be grouped into two different clusters. Thus, we observed a positive phalloidin activity for analogues **2**–**6**, which exhibited widely varying activities ranging from the most active (59% increase for **2**) to the least (33% for **4**). Although alanine scanning did not provide any valuable information regarding the preliminary structure–activity relationships, a different picture was observed for those analogues bearing the 2-NaI moiety. This second group of compounds (**7** and **8**) exhibited an inhibitory effect in our phenotypic assay. These findings therefore suggest that the presence of a naphth-2-yl side chain can impair the F-actin polymerization effect. The effect of a serine residue in position 3 of the tripeptides is not yet clear but it seems to provide a slight and additional reduction of F-actin polymerization when comparing peptides **2** and **7** with **6** and **8**, respectively. ILK inhibition has been widely related to disorganization of the actin cytoskeleton and polymerization, and this effect has been related to cancer biology.^13,34^ However, its selective adaptor/scaffolding activation has not been widely studied, and some studies propose that actin polymerization represents a new and promising approach for the treatment of CKD.^35,36^ As such, we selected peptides **2** and **3** for further characterizations due to their ability to increase F-actin polymerization. First, we examined the cellular viability for these compounds at different concentrations (Figure S4) and found that neither induced any significant toxicity. To determine whether the increased actin polymerization observed for compounds **2** and **3** was dependent on ILK, we silenced the ILK gen by using a specific siRNA (Figure S5).

When targeting ILK with a specific siRNA, a decrease in phalloidin activity with respect to the control was observed. In contrast, after treatment of HMC with compounds **2** and **3**, an increase in phalloidin content was observed by confocal microscopy, as expected for the proposed positive activity. A dramatic increase in phalloidin activity of about 100% was detected for **2**, and a similar trend was observed for **3** (up to 59% with respect to the control). In contrast, this phenomenon is minimized to near baseline values upon addition of a specific ILK siRNA in all cases (Figure 4). Similar results were not observed when actin polymerization was stimulated by forskolin, an adenylate cyclase agonist (Figure S6). As such, we concluded that the increased phalloidin activity observed with the tripeptides could be related to their ability to interact with ILK (Figure 4).

**Figure 4:**
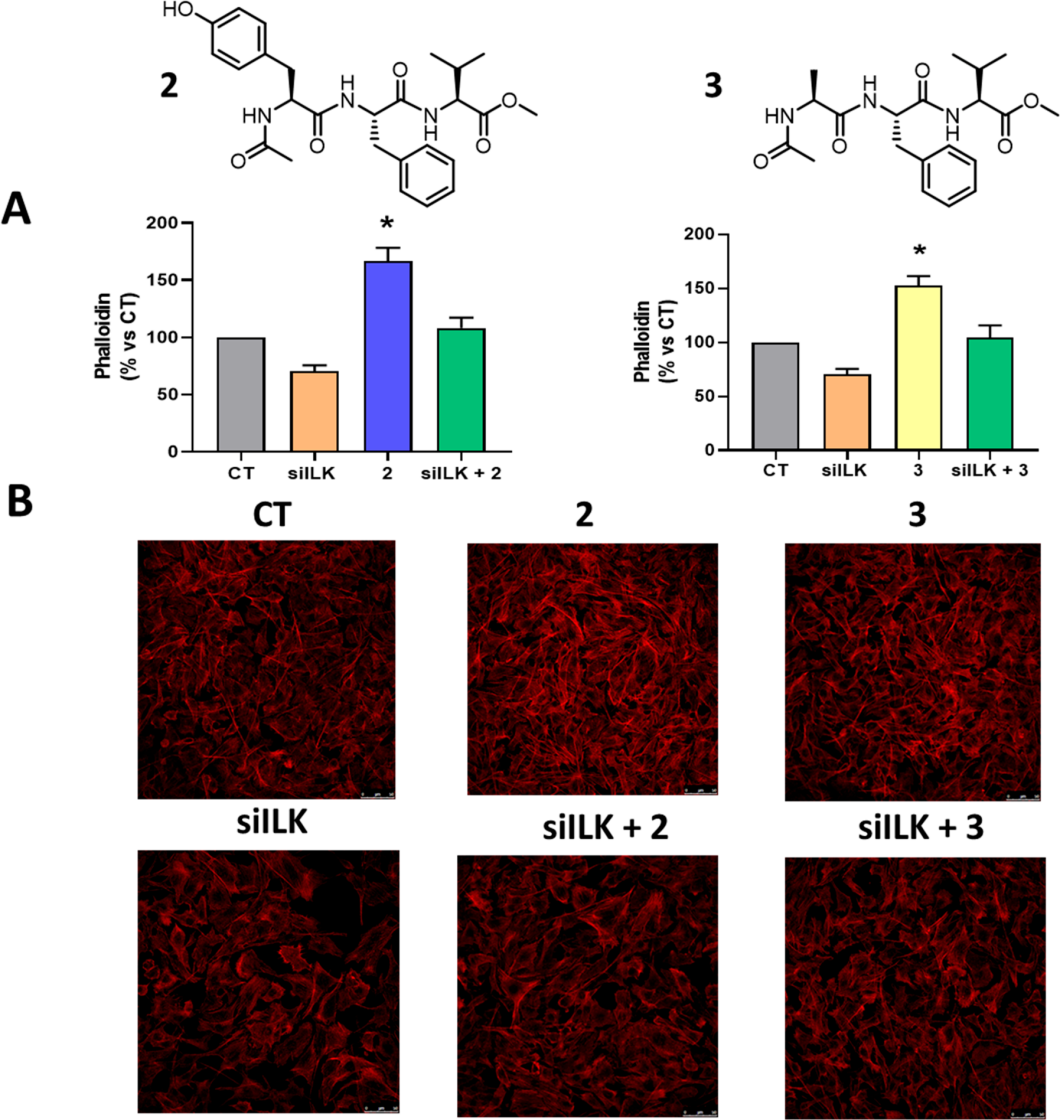
Evaluation of actin polymerization after integrin-linked kinase (ILK) inhibition with a siRNA of ILK (siILK). Human mesangial cells were transfected with siILK or scrambled siRNA as a transfection control, incubated in control conditions (CT, buffer 24 h) or with compounds **2** and **3** (50 μM, 24 h), stained with Alexa 568 phalloidin and examined by confocal microscopy. The amount of F-actin was measured in three independent assays. (A) Quantitative evaluation of actin polymerization (F-actin). Results are the mean ± SEM, expressed as percentage vs CT. *p < 0.05 vs CT. (B) Representative confocal image of Phalloidin immunostaining.

In conclusion, based on in silico approaches and a careful observation of the dimerization interface between ILK and α-parvin, we can propose a simplistic representation of a putative hot spot based on the predicted importance of Phe307 and surrounding residues (Tyr306, Gly305, and Val308) present in the α-parvin protein. Despite the lack of kinase control over ILK, these compounds are able to increase actin cytoskeletal content in an ILK-dependent mechanism, as shown for compounds **2** and **3**. Although peptides are not considered to be very interesting drug candidates, our findings may open up new possibilities for the modulation of ILK in vivo. These peptides are valuable chemical tools for the future design of peptidomimetics, chemical probes, or even drugs that activate the scaffolding properties of ILK in a highly selective manner. Furthermore, we are currently studying the dimeric interface using longer peptides to shed some light on the still uncertain role of the ILK−α-parvin interaction and its role in human cells for therapeutic purposes.

## Supporting Information Available

Molecular modeling methods, analytical data of all compounds, biological assays and peptide synthesis (PDF)

## Abbreviations

ILK: integrin-linked kinase
PDB: protein data bank
siRNA: small interference ribonucleic acid
MD: molecular dynamics
PINCH: particularly interesting new cysteine-histidine-rich protein
HCM: human mesangial cells
CKD: chronic kidney disease
2-NaI: β-(naphth-2-yl)-alanine
