# Supplementary material for: Tripeptides as Integrin-Linked Kinase Modulating Agents Based on a Protein–Protein Interaction with α-Parvin
Source: ACS Med Chem Lett. 2021 Jul 15;12(11):1656–62. doi: 10.1021/acsmedchemlett.1c00183 (PMC8591738; doi:10.1021/acsmedchemlett.1c00183)
Supplement: Supplementary file 1 — ml1c00183_si_001.pdf [file ml1c00183_si_001.pdf]

Tripeptides as Integrin-linked kinase modulating agents based on a protein-protein interaction with α-parvin

Javier Garcia-Marin,[a, d, e †, *] Mercedes Griera-Merino,[b, g] Alejandra Matamoros-Recio,[a, †] Sergio de Frutos,[b, d, f] Manuel Rodríguez-Puyol,[b, d, f] Ramón Alajarín,[a, d, e] Juan J. Vaquero,[a, d, e, *] Diego Rodríguez-Puyol.[c, d, f *]

[a] Departamento de Química Orgánica y Química Inorgánica and, [b] Departamento de Biología de Sistemas, Universidad de Alcalá, Alcalá de Henares 28805, Spain javier.garciamarin@uah.es

[c] Fundación de Investigación Biomédica, Unidad de Nefrología del Hospital Príncipe de Asturias y Departamento de Medicina y Especialidades Médicas, Universidad de Alcalá, Alcalá de Henares 28805, Madrid Spain.

[d] Instituto Ramón y Cajal de Investigación Sanitaria (IRYCIS) Ctra. Colmenar Viejo, km. 9100, Madrid 28034, Spain

[e] Instituto de Investigación Química Andrés Manuel del Río (IQAR), Universidad de Alcalá, Alcalá de Henares 28805, Spain

[f] Fundación Renal Iñigo Álvarez de Toledo (FRIAT) y REDinREN del Instituto de Salud Carlos III, 28029, Madrid, Spain

[g] Graphenanano Medical Care, S.L., Yecla 30510, Spain

Supporting information

Table of contents:

1. **Molecular modeling: Hot spot, Molecular dynamic**
2. **Analytical data (HPLC-HRMS) for the peptides synthesized and tested in this study**
3. **Biological assays and methods**
4. **Experimental procedures for the synthesis of 1 to 8**
5. **NMR spectra experiments for representative key compounds 2 and 3**
6. **Bibliography**

1 Molecular modeling

1.1 Hot spot calculations

For the *in silico* identification of hot spots two different approaches were used. First, ILK kinase domain (PDB 3KMW, Chain A), was subjected to FTMap server (http://fmap.bu.edu/) in PDB format. Results were retrieved in PyMOl session format and analysed.[1] FTMap algorithm docks small organic probe molecules with different size, shape and polarity on the surface of the protein submitted. Then, it finds the most favourable positions for each probe type, clusters them, and ranks the clusters based on its energy. Those regions that bind several different probe clusters are selected as consensus clusters or consensus sites and they represent putative binding *hot spots*.

On the other hand, HotPoint method was also used for the *hot spot* identification. [2] This approach determines computationally hot spots based on pair potentials and solvent accessibility of interface residues. [3] The accession code 3KMW was introduced into the server and then, results were analysed by means of post-scripts retrieved from web.

A

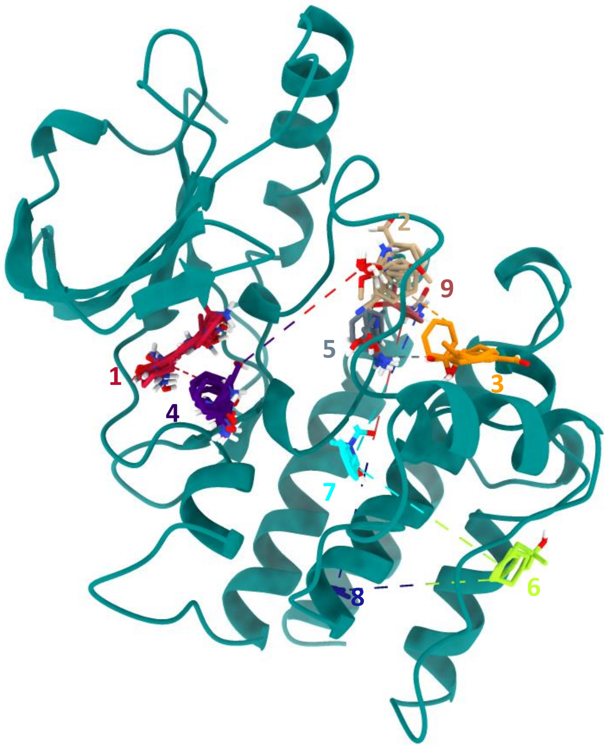

B)

Residue Number	Residue Name	Chain	RelCompASA	RelMonomerASA	Potential	Prediction
225	R	A	36.25	84.55	12.10	NH
348	G	A	19.94	114.33	13.01	NH
349	R	A	16.67	56.49	16.49	NH
350	M	A	21.45	50.75	27.53	NH
351	Y	A	28.02	45.03	15.13	NH
353	P	A	0.35	2.84	12.32	NH
361	L	A	0.00	21.03	37.51	H
362	Q	A	32.43	55.61	17.04	NH
363	K	A	44.08	48.39	4.11	NH
364	K	A	42.60	68.59	4.05	NH
397	N	A	25.55	44.28	4.70	NH
398	M	A	0.05	72.32	38.37	H
399	E	A	10.10	40.37	14.36	NH
401	G	A	0.00	15.19	13.82	NH
402	M	A	9.41	51.22	42.67	H
403	K	A	15.12	31.96	13.77	NH
406	L	A	51.39	68.30	25.92	NH
278	K	B	28.10	52.60	9.00	NH
279	L	B	9.51	47.70	34.16	H
298	L	B	1.16	17.09	60.96	H
301	G	B	0.54	8.01	14.69	NH
302	L	B	6.90	26.28	44.28	H
305	G	B	36.74	89.36	8.42	NH
306	Y	B	1.70	39.86	17.91	NH
307	F	B	0.00	75.41	35.04	H
308	V	B	0.15	7.24	35.20	H
309	P	B	5.81	40.39	4.12	NH
310	L	B	12.46	53.13	30.94	H
311	H	B	28.03	91.15	3.94	NH
312	S	B	28.87	42.74	10.55	NH
315	L	B	28.04	41.91	33.75	NH
332	E	B	43.40	55.85	8.75	NH
333	L	B	1.98	11.67	43.21	H
335	Q	B	18.50	34.76	12.51	NH
336	D	B	40.20	84.39	10.19	NH

Figure S 1. *A) Cluster probes (hot spot) detected by FTMap. B). HotPoint table of results retrieved by the web server. NH = Hot spot, NH = Non hot spot.*

1.2 Molecular dynamics:

PDB files were upload to CHARMM-GUI and prepared for molecular dynamics simulations. [4] Tripeptide starting geometries were generated directly from the chain B (α-parvin) of the PDB 3KMW using PyMol (The PyMOL Molecular Graphics System, Version 1.7 Schrödinger, LLC). Using the *Solution Builder* module, protein, ATP, and Mg^{2+} ion names were changed to those used in CHARMM36m forcefield and psf topology files were generated. [5] Then, the system was immersed in a rectangular water box of TIP3P water molecules extended 10 Å from protein solute. Afterwards, 3 Na^{+} ions were added to neutralize the system. Archives containing PDB coordinates, topology and forcefield parameters were downloaded together with production scripts. The MD protocol included an energy minimization with conjugate gradients for 2000 cycles and equilibration at 298 ^{o}C in the NVT ensemble for 1 ns applying positional harmonic restrains over protein atoms (k = 10 kcal mol^{-1}Å^{-2}). After this, MD production was performed without any restrain using NAMD 2.1[6] in a GPU NVIDIA for 50 ns with the Nosé-Hoover Langevin piston pressure control through their CUDA implementations on a Windows 10 workstation equipped with a GPU NVIDIA GeForce GTX 650 Ti BOOST.

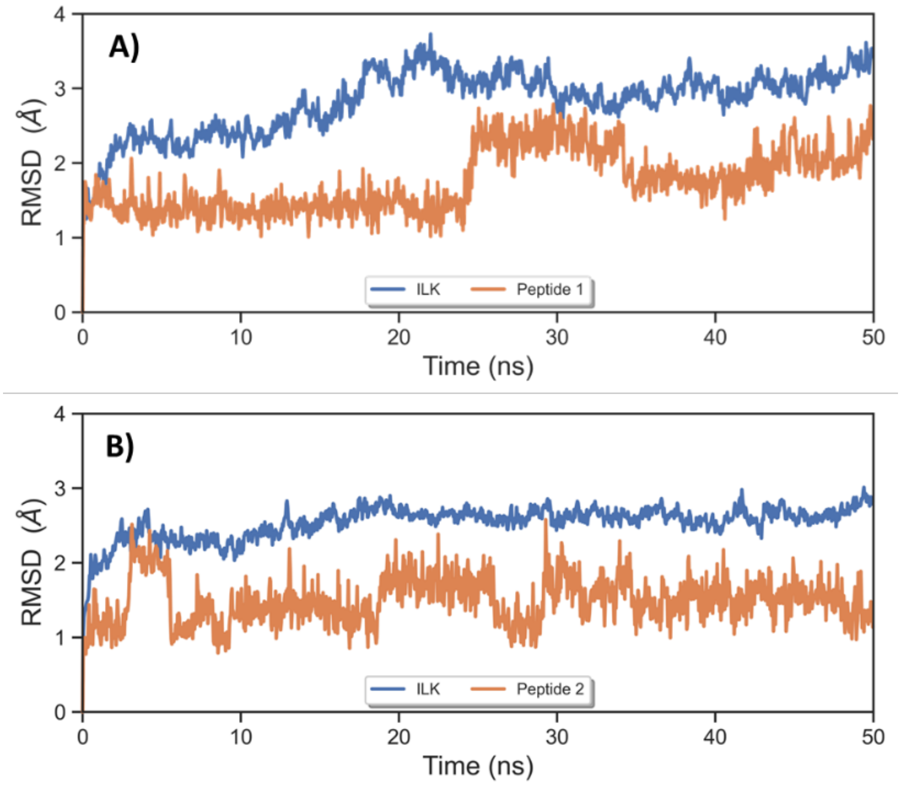

Figure S 2. *RMSD plots of compounds **1** and **2** bound to ILK kinase domain respectively.*

2 Analytical data for the peptides synthesized and tested in this study

(1) Ac-Tyr-Phe-Val-OH

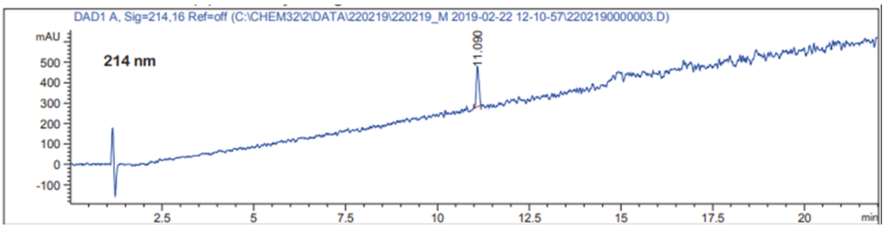

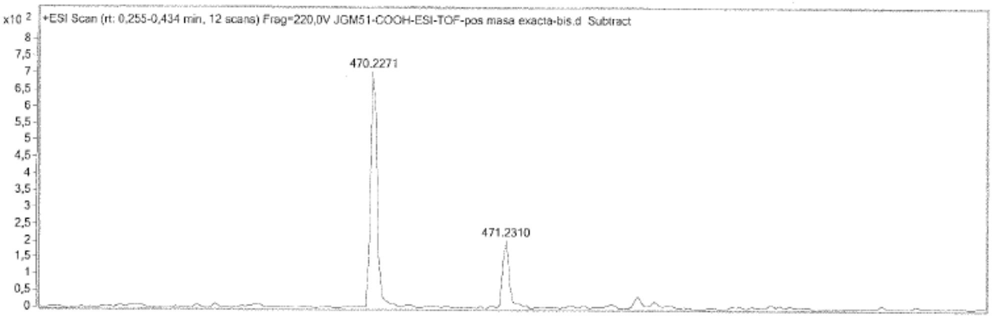

(2) Ac-Tyr-Phe-Val-CH_3

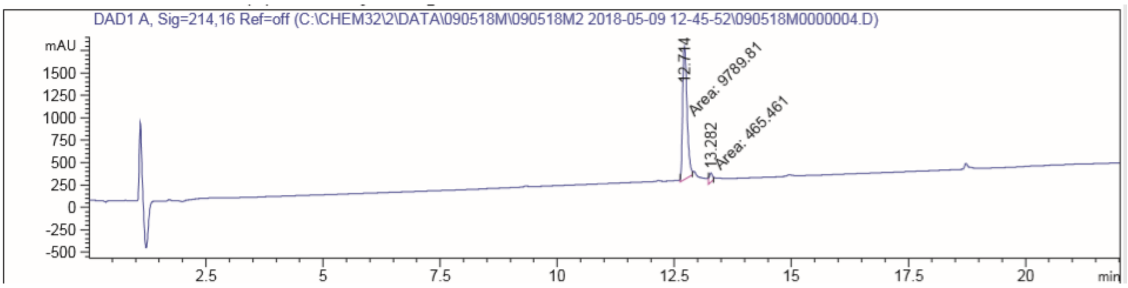

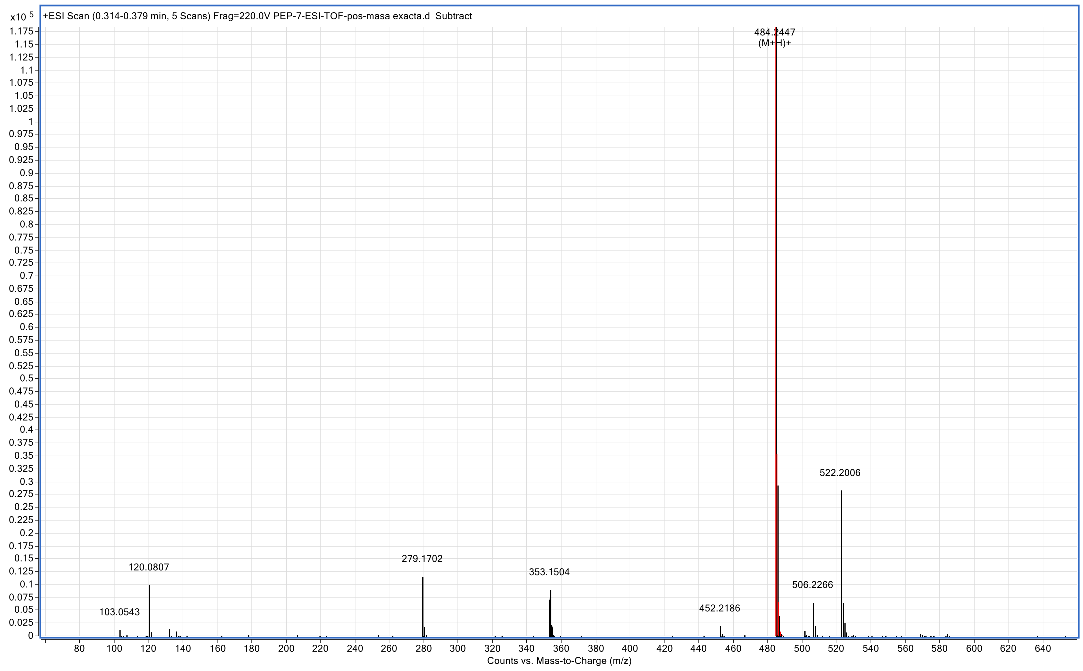

(3) Ac-Ala-Phe-Val-CH$_3$

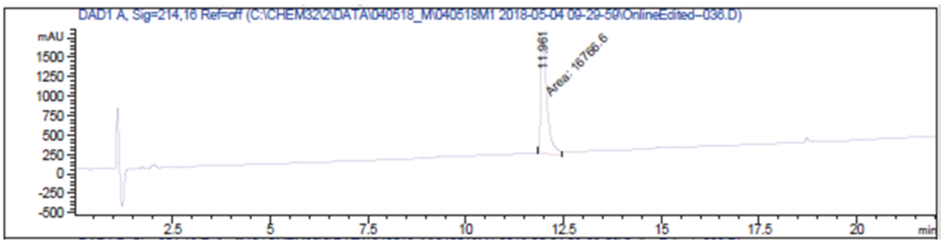

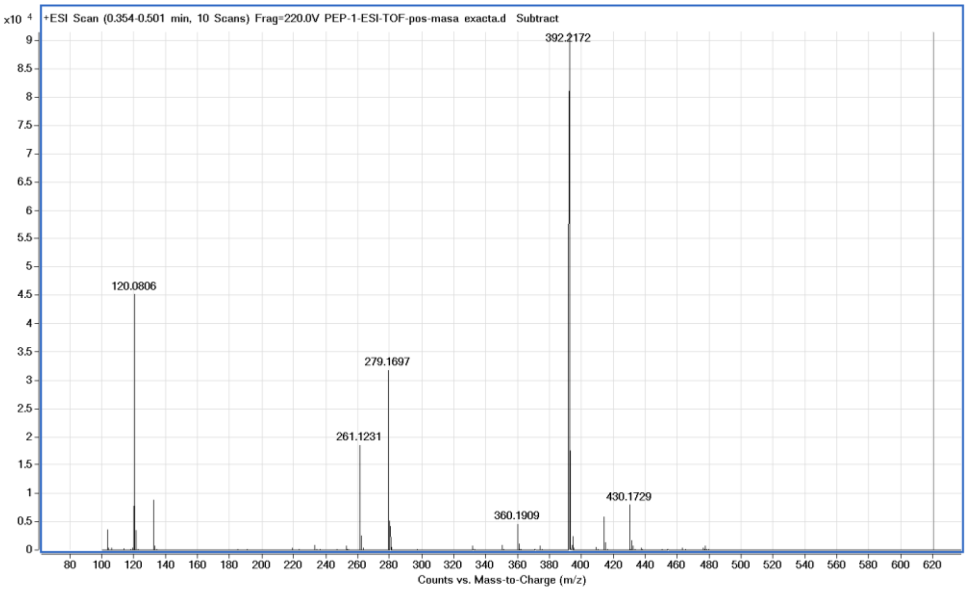

(4) Ac-Tyr-Ala-Val-OCH$_3$

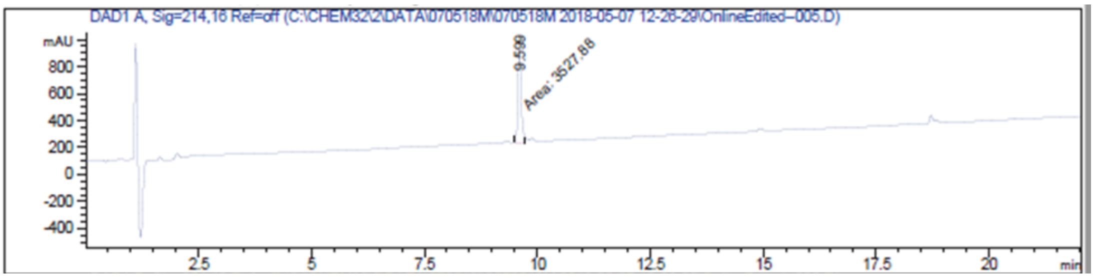

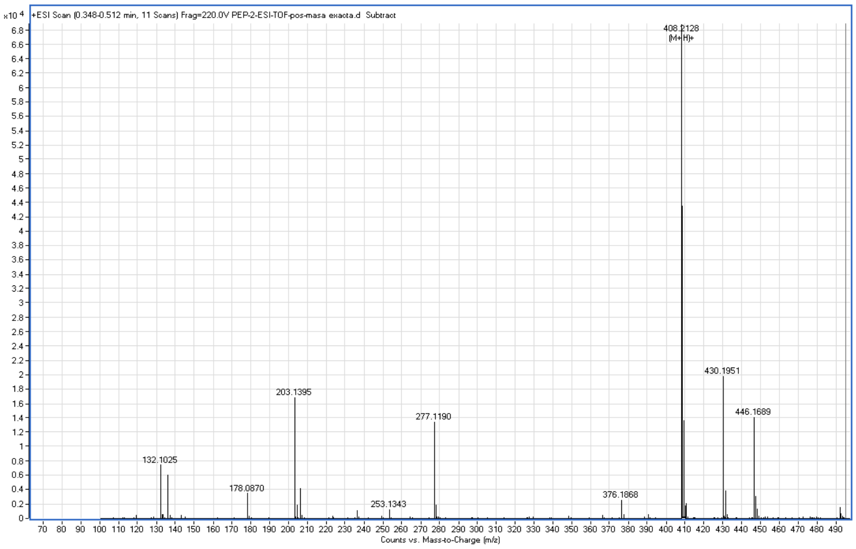

(5) Ac-Tyr-Phe-Ala-OCH_3

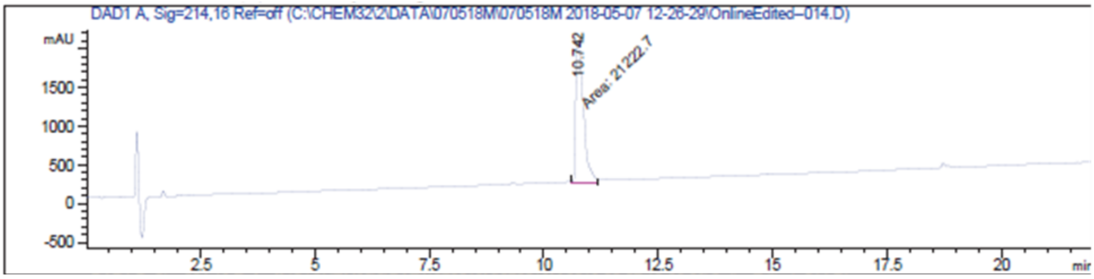

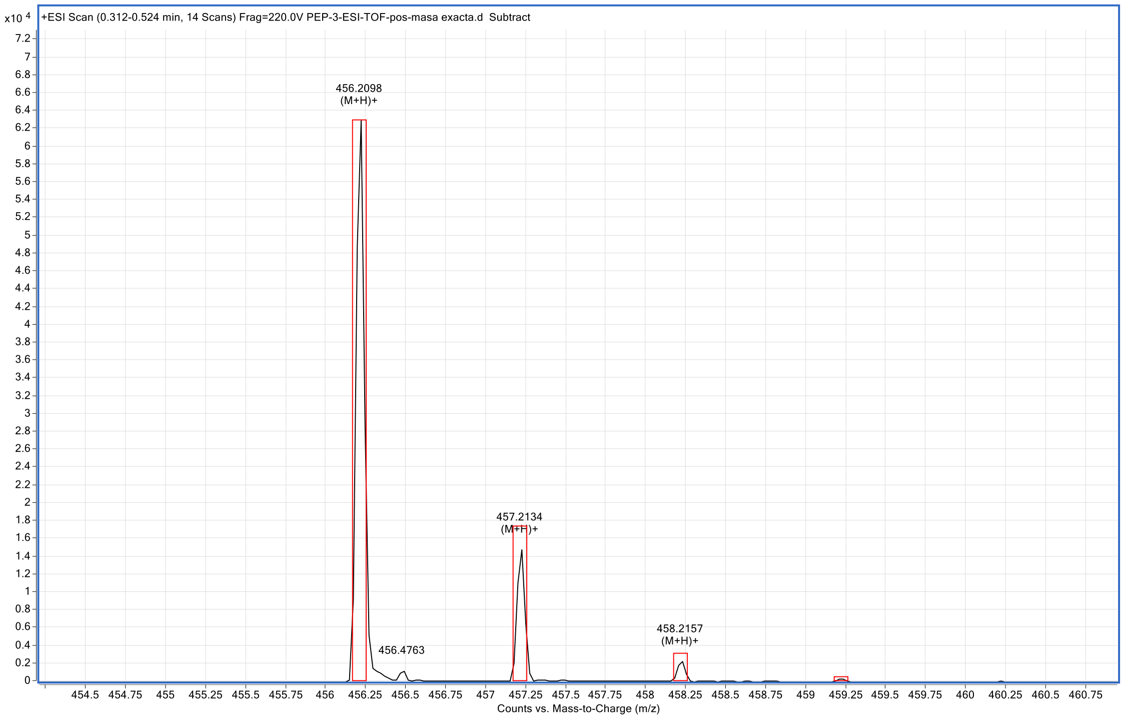

(6) Ac-Tyr-Phe-Ser-OCH$_3$

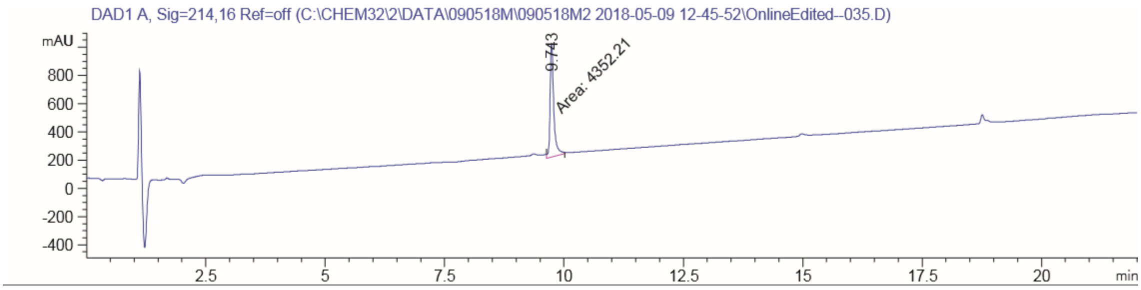

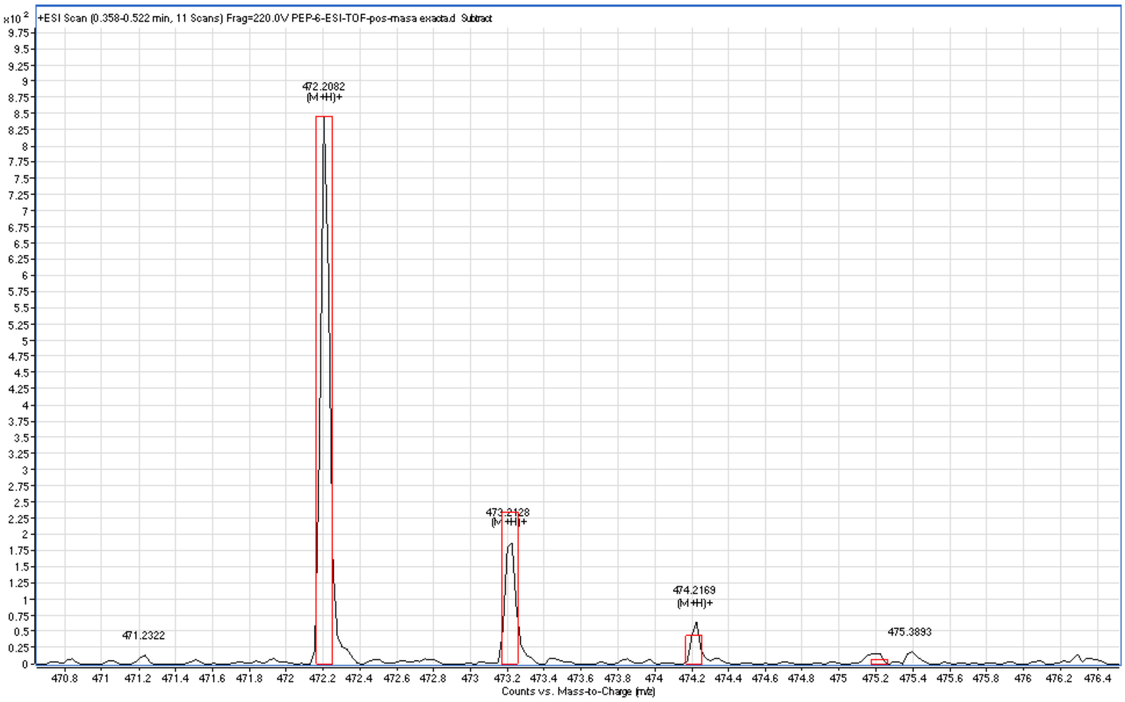

(7) Ac-Tyr-22NaI-Val-OCH$_3$

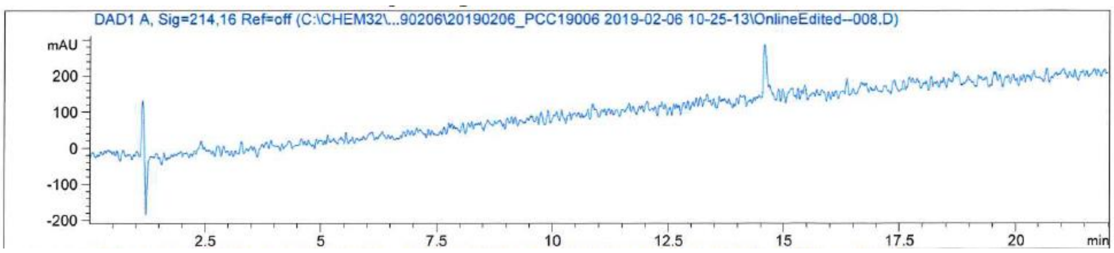

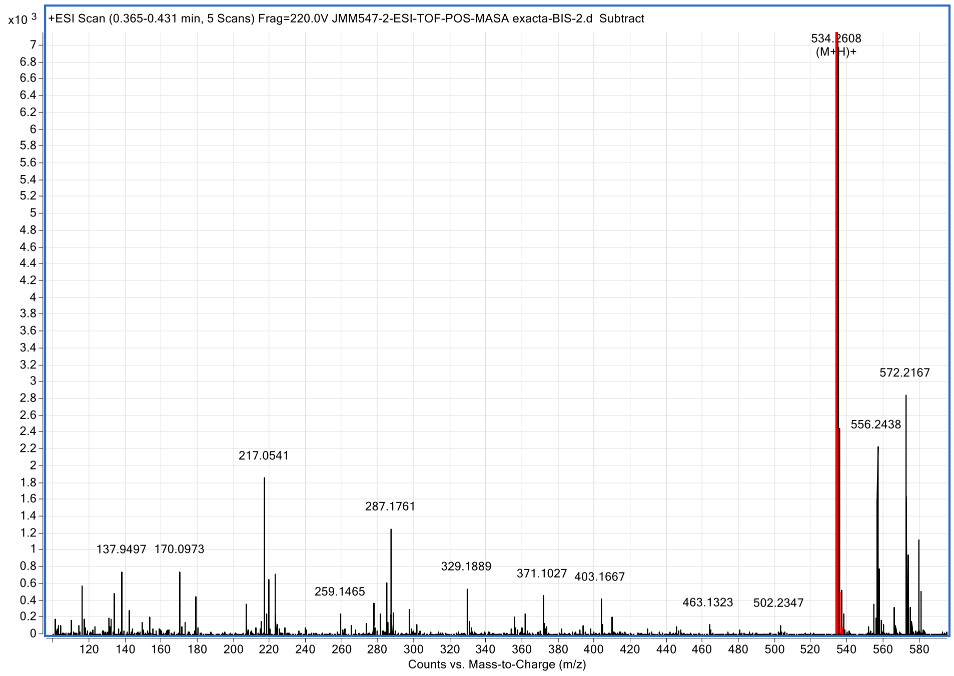

(8) Ac-Tyr-22NaI-Ser-OCH$_3$

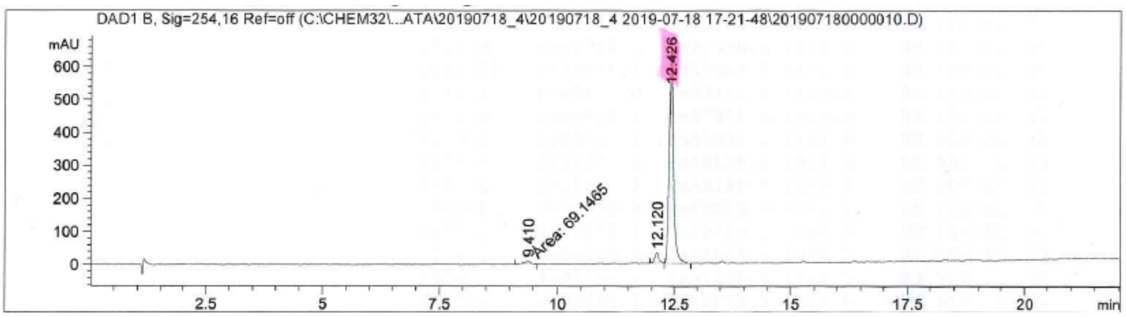

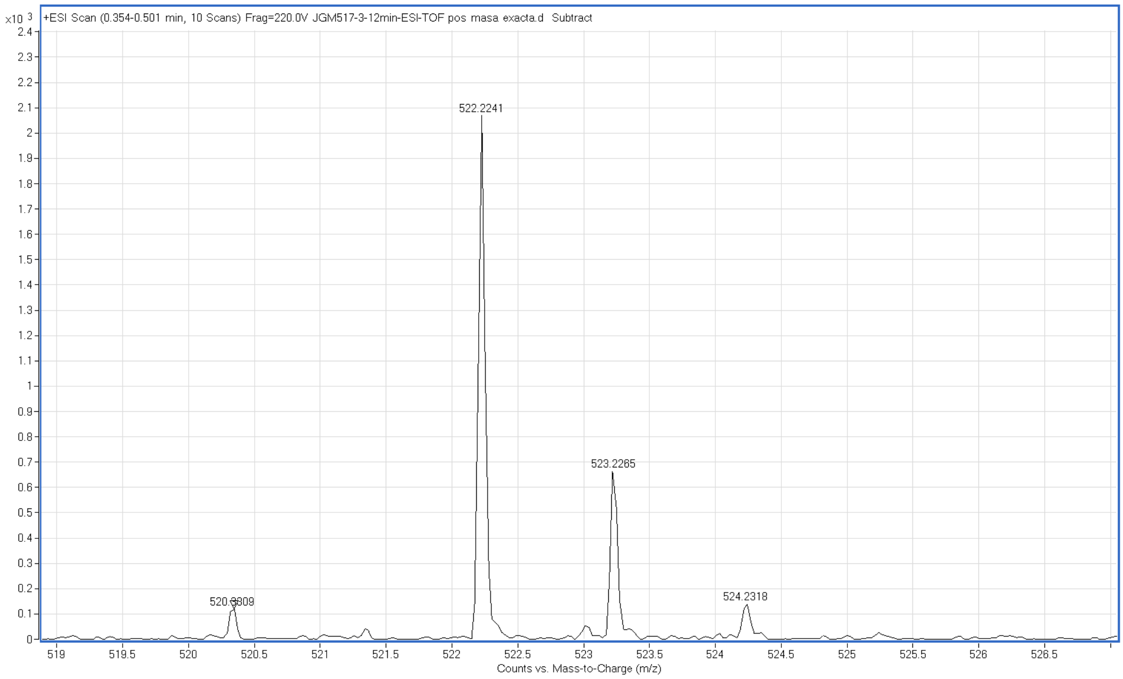
x10 3
+ESI Scan (0.354-0.501 min, 10 Scans) Frag=220.0V JGM517-3-12min-ESI-TOF pos masa exacta.d Subtract
522.2241
523.2265
524.2318
520.3309
Counts vs. Mass-to-Charge (m/z)

3 Biological assays and methods

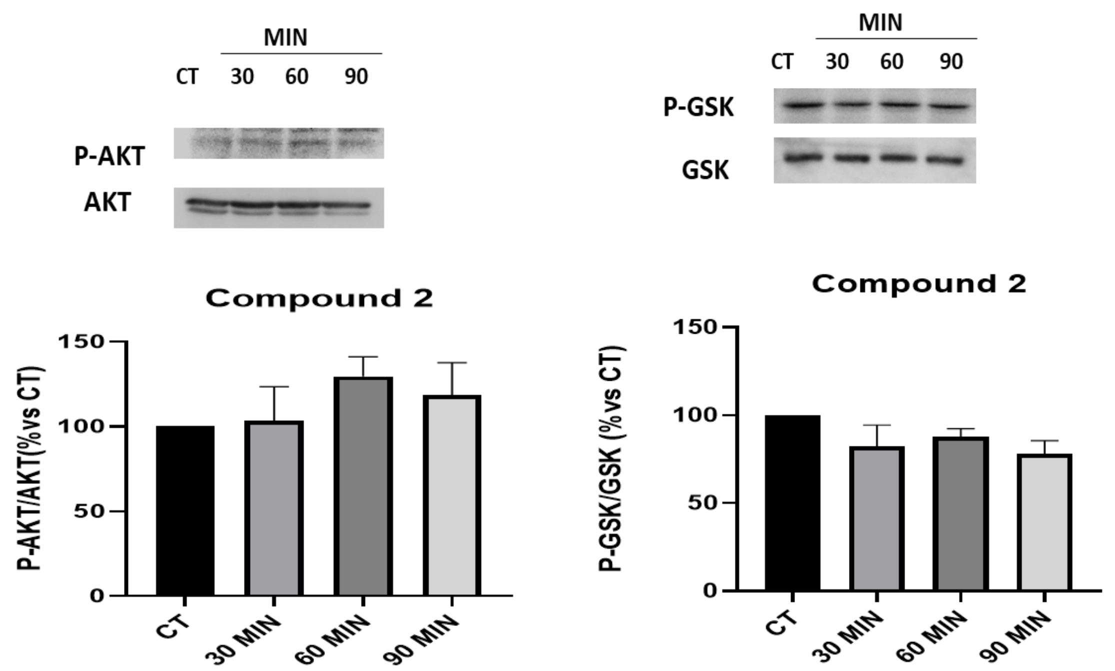

Figure S 3 : *Analysis of the kinase activity of ILK in human mesangial cells. Cells were incubated with buffer (CT) or 50 µM Compound 2 for different periods of time, and AKT phosphorylation at Ser-473 (pAKT) and GSK phosphorylation at Ser-9 (P-GSK) were evaluated by immunoblot. The upper panel of the figure shows a representative immunoblot, whereas the lower panel shows the densitometric analysis of blots normalized against total AKT and GSK. The results are expressed as a percentage of control cells and are the mean* + *SEM of 5 independent experiments.*

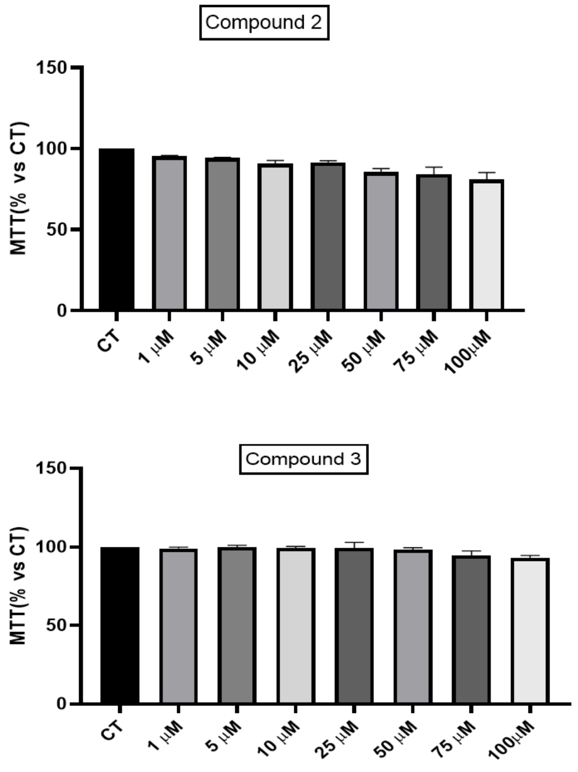

Figure S 4 Effects of Compound 2 and 3 on the reduction of 3-(4,5-dimethylthiazol-2-yl)-2,5-diphenyl tetrazolium bromide (MTT). Human mesangial cells were incubated with different concentrations of the compounds at 37ºC for 24h, and MTT assay was performed. The results are expressed as the percentage of the basal level and represent the mean + SEM of 4 independent experiments.

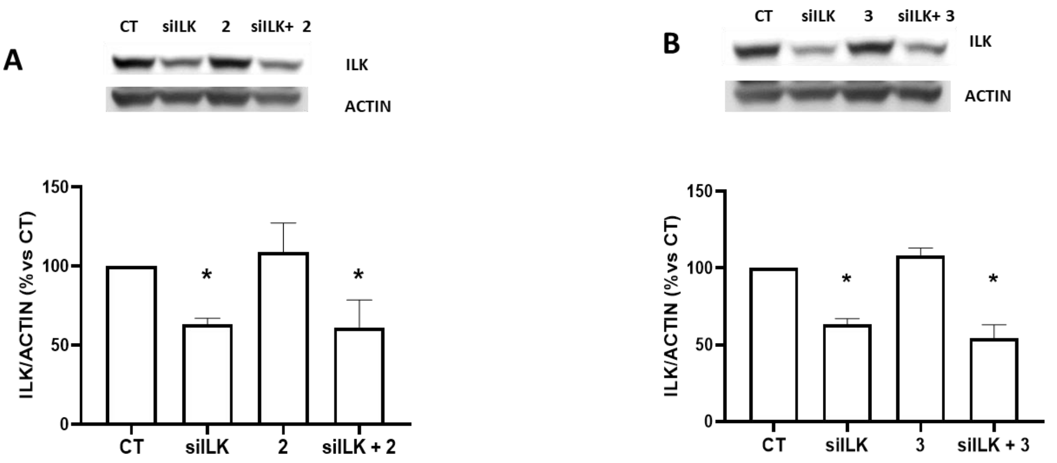

Figure S 5. *Evaluation of the integrin-linked kinase (ILK) cellular content after cell incubation with siRNA against ILK (siILK). Human mesangial cells were transfected with siILK or scrambled siRNA as a transfection control (CT) 24h and co-treated with 50 µM of compound **2** (panel A), **3** (panel B) or vehicle (buffer) for another 24h. ILK content was measured by western blot and figures show a representative image and the quantitative evaluation of the ILK densitometries normalized against actin content. Results are the mean ± SEM of three independent experiments, expressed as percentage vs CT. *p<0.05 vs CT.*

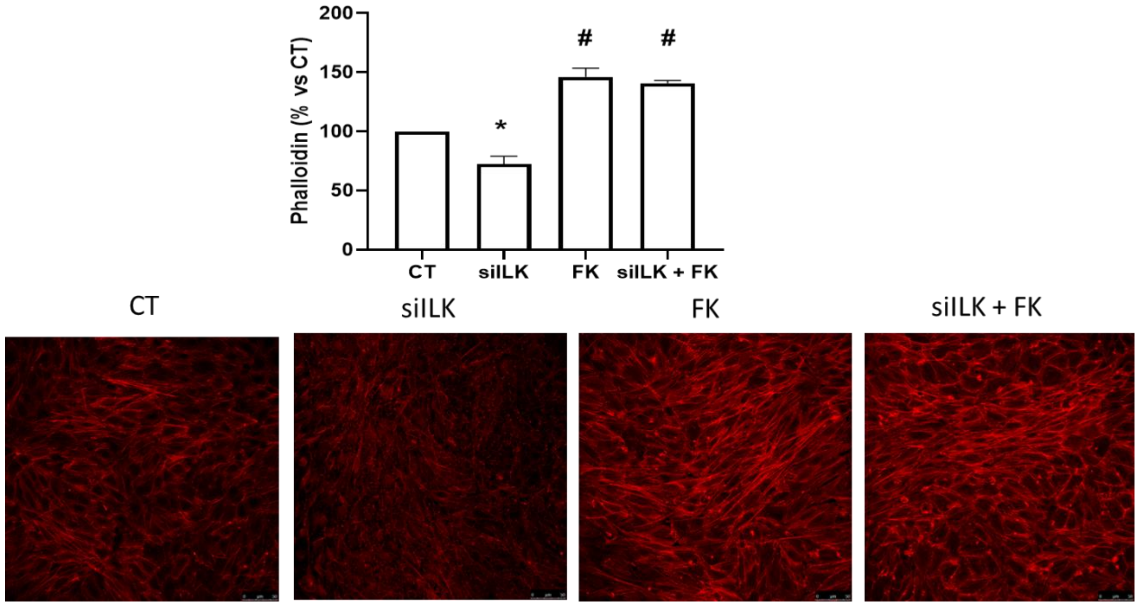

Figure S 6. *Evaluation of actin polymerization after integrin-linked kinase (ILK) inhibition with siRNA against ILK (siILK). Human mesangial cells were transfected with siILK or scrambled siRNA as a transfection control (CT) 24h and co-treated with forskolin (FK, adenylate cyclase activator, 10 mM) or vehicle (buffer) for 1 h. Cells were stained against actin polymerization (F-actin) with Alexa 568 phalloidin and examined by confocal microscopy. Panel A: Quantitative evaluation of F-Actin. Panel B: Representative confocal images of phalloidin stainings. Results are the mean ± SEM of three independent experiments expressed as percentage vs CT. *p<0.05 vs CT. #p<0.05 vs CT and siILK.*

.

3.1 Cell culture

Human mesangial cells (HMC) were cultured according to previously described procedures [7]. Briefly, portions of macroscopically normal cortical tissue were obtained from human kidneys immediately after nephrectomy. Isolated glomeruli were treated with collagenase type IA and plated in plastic culture dishes. They were maintained in RPMI 1640 supplemented with 10% fetal bovine serum, l-glutamine(1 mM), penicillin (0.66 g/mL), streptomycin sulfate (60 g/mL),and buffered with HEPES and bicarbonate, pH 7.4, in a 5% CO^2 atmosphere. All Reagents were from Sigma-Aldrich (Merck KGaA, Darmstadt, Germany) The identity of the cells was confirmed by morphologic and functional criteria. Culture media were changed every 2 days. When the cells reached confluence, they were subcultured at a ratio of 1:4 using the same incubation medium. The cells were serum-deprived for 24 h before the treatments.

3.2 MTT viability test

Toxicity tests were achieved in HMC as previously described. [8] Briefly, cells were seeded in 24-well plates and, once they reached confluency, they were deprived and treated with the compounds for 24 h. RPMI was supplemented with 3-(4,5-dimethylthiazol-2-yl)-2,5-diphenyl tetrazolium bromide (MTT, Merck KGaA, Darmstadt, Germany) (0.5 mg/mL final concentration) and incubated at 37°C. After 4 h, The medium was removed, and the purple formazan crystals formed were then dissolved by adding 500 µL of dimethyl sulfoxide (DMSO, Merck KGaA, Darmstadt, Germany) and mixed effectively by pipetting up and down. Spectrophotometric absorbance of the purple blue formazan dye was measured using Multimode Plate Reader (Perkin Elmer) at 570 nm. Optical density of each sample was compared with control optical density.

3.3 Transfection with siRNA

HMC were lipofectamine-transfected with a mixture of three specific siRNAs against ILK or unspecific, scramble siRNAs) as a control as previously described. [9] siRNAs were purchased at Santa Cruz Biotechnologies, Dallas, TX. A mixture of the three specific oligonucleotides or scramble siRNAs at 20 nM concentration were mixed with 3 µL of Lipofectamine 2000, in 200 µL of Opti-MEM (both reagents from Thermo Fisher Scientific Waltham, MA, USA) and incubated at 37 °C for 20 min. Then, the mixture was added to HMC at 70% of confluence in 800 µL of Opti-MEM and incubated overnight at 37 °C. 72 hours after transfection, cells were treated with the compounds for 24 additional hours.

3.4 Protein extraction and Western blot analysis

After the treatments with the compounds, HMC were washed in PBS and solubilized in lysis buffer (10 mM Tris-HCl, pH 7.6; 1% Triton X-100; 1 mM EDTA; 0.1% sodium deoxycholate) supplemented with protease and phosphatase inhibitors (Complete and PhosSTOP, Roche, Basel, Switzerland). Protein concentrations were determined by DC-Protein Assay (Bio-Rad, Hercules, CA, USA). Equal amounts were separated on SDS-polyacrylamide gels and transferred to 0.2µm-PVDF membranes (Bio-Rad, Hercules, CA, USA). Membranes were blocked, incubated with primary antibodies against GLUT4 (Santa Cruz, Dallas, TX, USA), P-GSK-3β(Ser9), GSK-3β, P-AKT(Ser473), AKT (Cell Signaling Technology, Inc., Danvers, MA, USA), and secondary antibodies (Dako, Glostrup, Denmark) afterwards, as previously described. [10] Immunoblots were detected by chemiluminescence (Pierce ECL Western Blotting Substrate, Thermo Fisher Scientific Waltham, MA, USA) and imaged with ImageQuant LAS 500 System (General Electric Healthcare, Little Chalfont, Buckinghamshire, United Kingdom). Densitometries were measured using ImageJ software (NIH, USA). GSK-3β and AKT proteins were used as endogenous control.

3.5 Immunofluorescence microscopy

For intracellular localisation and quantitative counting of filamentous actin (F-actin), immunofluorescence determination of F-actin was achieved in HMC after the treatments, as previously described [11]. Briefly, HMC were plated on coverslips. After the treatment with the compounds or forskolin, cells were fixed with 4% paraformaldehyde for 30 min and permeabilised with 0.05% Triton-X-100 in phosphate buffered saline (PBS) for 10 min. Cells were incubated with 2.5% bovine serum albumin (BSA) on PBS at room temperature for 1 h and washed afterwards. Reagents from Merck KGaA, Darmstadt, Germany. For intracellular localisation and quantitative counting of filamentous actin (F-actin), cells were incubated with 0.1 µg/mL Alexa 568-phalloidin (Molecular Probes, Thermo Fisher Scientific Waltham, MA, USA) for 45 min at room temperature. Coverslips were washed with PBS and distilled H_2O before to ad Vectashield mounting medium that includes nuclear colouring (Vector Laboratories, UK). Confocal images of the coverslips were obtained with a Zeiss LSM 510 Meta confocal laser scanning head attached to a Zeiss. Images were exported and F-actin was quantified with ImageJ software (NIH, USA).

2 Experiental procedures for the synthesis of 1 to 8.

All reagents were acquired from the following commercial sources and used without further purifications: Merck, ACROS, Novabiochem, Fluorochem and Alfa Aesar. Solvents were purchased from Scharlab. ^{1}H and ^{13}C-NMR spectra were recorded on a either a Varian Mercury VX-300, Varian Unity 300 or Varian Unity 500 MHz spectrometer at room temperature in the deuterated solvent stated. Chemical shifts (δ) are quoted in parts per million (ppm) and referenced using the water peak or solvent residual peak as an internal reference (δ_H = 3.31 ppm and δ_C = 79.0 ppm for CD_3OD). Multiplicities are denoted as singlet (s), doublet (d), triplet (t), quartet (q), apparent (ap) and multiplet as (m). The abbreviation br denotes a broad resonance peak. Coupling constants (*J*) were recorded as Hert (Hz).

High-resolution analysis (HRMS) were performed on an Agilent 6210 time of flight LC/MS using electrospray (ESI) as ion source and mobile phase of ACN/H_2O 75:25 with 0.1% TFA as additive.

Semipreparative HPLC

**Column:** KROMAPHASE C18	
**Column temperature**	Room temperature
**Phases**	A= Formic acid 0.1% (v/v)<br>B= CH_3OH with formic acid 0.1% (v/v)
**Flow rate**	20 mL/min
**Gradient**	10-100% B in 20 min
**Injection volume**	10 mL
HPLC dynamax VARIAN (equipped with two binary pumps, manual injector, UV-VIS detector)	

Kaiser Test

The Kaiser or ninhydrin test for the detection of primary amines was carried out by taking a small amount of dried resin in a small glass tube. To the dried resin were added 2 drops of 5% (w/v) ninhydrin in ethanol, 2 drops of 80% (w/v) phenol in ethanol, and 2 drops of 20 μM potassium cyanide (KCN) in pyridine (0.01M). The tube was then heated for a few seconds at ∽ 120 °C. The test is used routinely to monitor the presence of free amine after deprotection (dark blue color, positive) and the completeness of the amino acid coupling step (yellow color, negative test).

Chloranil Test

The Chloranil test for secondary amine was conducted by taking a small amount of dried resin in a small glass tube. Four drops of 2% p-chloranil (w/v) in toluene were added to the dried resin and next, 10 drops of acetone. The tube was then agitated for few seconds at room temperature. No coloration develops in the beads supporting an Fmoc-protected *peptide,* while dark green coloration was observed after Fmoc removal.

Positive Kaiser Test	Negative Kaiser Test	Positive Chloranil Test
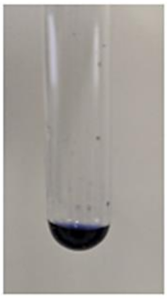	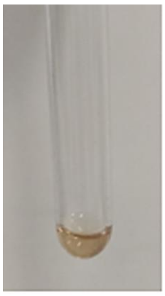	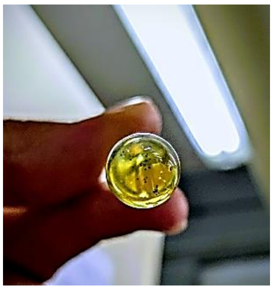

2.1 General Methods for Solid Phase Peptide Synthesis

All peptides were synthesized using standard Fmoc-based solid phase peptide synthesis (SPPS) procedures on 2-chlorotrityl chloride resin (1.55 - 1.27 mmol/g) and using commercially available Fmoc-protected amino acids. The synthesis was performed in a 5 mL plastic syringe provided with caps and frit (25 µm pore size, Multi Syn Tech GmbH). Reactors were shaken using IKA RW 20 digital mixer at 73 rpm and set horizontally in a Heidolph Reax 2 overhead shaker. Polypropylene reactors were filtered using a VacMaster multiport vacuum manifold coupled to a water-jet pump.

2.1.1 Resin Swelling

2-Chlorotrityl chloride resin (100 mg) was placed in a clean, dry plastic syringe with DCM (30 mL/g) and capped. The reactor was shaken with DCM (20 mL/g) for 5 min and then, DCM was removed with the vacuum manifold.

2.1.2 Initial Resin Loading

Fmoc-aa-OH (1.5 equiv) was dissolved in dry DMF (30 mL/g of resin) and DIPEA (3 equiv) was added. The final solution was mixed with 2-chlorotrytl chloride resin (100 mg). The suspension was shaken for 1.5 h at room temperature. After this, the reaction was quenched with MeOH (0.8 mL/g of resin) and shaken for further 30 min. Then, the mixture was filtered off and subsequently washed with DMF, MeOH, DCM (30 mL/g, 3 x 3 min).

2.1.3 Fmoc Removal

The Fmoc-aa-resin or Fmoc-peptide-resin (100 mg) of was treated with 20% piperidine in DMF (3 mL) and stirred in a rotational shaker for 30 min at room temperature. Then, the mixture was filtered off and subsequently washed with DMF, MeOH, DCM (30 mL/g, 3 x 3 min). After this, a Kaiser test was carried out to determine the completion of the reaction (blue, positive).

2.1.4 Loading Test

Resin loading was verified by measuring the absorbance of the piperidine-dibenzofulvene adduct formed after Fmoc removal of the first supported amino acid. A 100 µL aliquot of the Fmoc-cleavage cocktail was taken prior reactor filtration. This was diluted into 10 mL of DCM and then, the absorbance of the mixture at 301 nm was measured by using a UV-Vis spectrophotometer to estimate the level of Fmoc removal, which correlates to the yield of the anchoring step and to the loading of the new resin. The first residue attachment was estimated from the following equation:

$$Substitution\ (\mathrm{mmol/g}) = \frac{Abs}{7800} 3000$$

Where Abs is the absorbance at 301 nm, 7800 is the extinction coefficient (ε) of the piperidine-divenzofluorene adduct and 3000 is a diluting factor.

2.1.5 General Resin Elongation (Standard Fmoc/tBu SPPS)

Fmoc-aa-OH (3 equiv) was dissolved in dry DMF (30 mL/g of resin) then, the corresponding coupling agent (3 equiv) and DIPEA (6.5 equiv) were added and the solution stirred for a few seconds. The final solution was mixed with preswelled NH_2-aa-O-resin or NH_2-peptide-O-resin in a rotational shaker for 3 h at room temperature.

Then, the mixture was filtered off and subsequently washed with DMF, MeOH, DCM (30 mL/g, 3 x 3 min). Then, a Kaiser test was performed to determine the completion of the reaction (yellow, negative).

2.1.6 *N*-Terminus acetylation

Peptide-NH_2 resin was mixed with DCM/acetic anhydride solution (1:1, 30 mL/g of resin) and shaked for 30 min at room temperature. Then, the mixture was filtered off and subsequently washed with DMF, MeOH, DCM (30 mL/g, 3 x 3 min). After this, a Kaiser test was carried out to determine the completion of the reaction (yellow, negative).

2.1.7 Resin cleavage and lateral chain deprotection

A) *N*-acetil-peptide resin was mixed with a TFA/TIPS/H_2O 9:0.5:0.5 solution (3 mL, 30 mL/g of resin) and shaken for 3 h at room temperature. Subsequently, the mixture was filtered, solvents were removed under vacuum and the residue was precipitated in cold ether and sonicated to afford a white solid that was sonicated, centrifuged and washed twice with diethyl ether to yield the final product.

B) *N*-acetil-peptide resin was mixed with 4M HCl in 1,4-dioxane/TIPS/MeOH solution 9:0.5:0.5 (30 mL/g of resin) and shaked for 5 h at room temperature. Subsequently, the mixture was filtered, solvents were removed under vacuum and the residue was precipitated in diethyl ether to afford a white solid that was sonicated, centrifuged and washed twice with diethyl ether to yield final product.

Ac-Tyr-Phe-Val-OH (1)

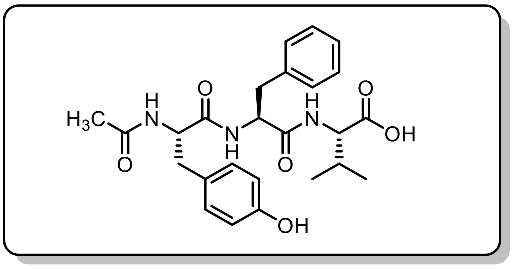

As described in the general peptide synthesis method, starting from 2-chlorotrityl chloride resin (1.47 mmol/g), HTCU as coupling agent, and using the general cleavage procedure **A**, **1** was obtained as a white solid (61 mg, 89%) with 100% purity.

^{1}H-NMR (500 MHz, CD_3OD): δ 7.27 – 7.12 (m, 5H, ArH Phe), 6.96 (d, *J* = 8.5 Hz, 2H, Ar-H2 and Ar-H6 Tyr), 6.63 (d, *J* = 8.5 Hz, 2H, Ar-H3 and Ar-H5 Tyr), 4.68 (dd, *J* = 8.5, 5.7 Hz, 1H, Hα Phe), 4.49 (dd, *J* = 9.0, 5.5 Hz, 1H, Hα Tyr), 4.29 – 4.22 (m, 1H, Hα Val), 3.12 (dd, *J* = 13.9, 5.5 Hz, 1H, CH_{2a} Phe), 2.95 – 2.86 (m, 2H, CH_{2a} Tyr and CH_{2b} Phe) 2.66 (dd, *J* = 14.1, 9.1 Hz, 1H, CH_{2b} Tyr), 2.12 (m, 1H, CH Val), 1.86 (s, 3H, $COCH_3$), 0.94 (d, *J* = 2.7 Hz, 6H, 2xCH_3 Val).

^{13}C-NMR (125 MHz, CD_3OD): δ 174.36 (CO), 173.46 (CO), 173.29 (CO), 173.05 (CO), 157.20 (Ar-C4 Tyr), 138.18 (Ar-C1 Phe), 131.17 (Ar-C2 and C6 Tyr), 130.45 (Ar-C3 and C5 Phe), 129.37 (Ar-C2 and C6 Phe), 127.70 (Ar-C4 Phe), 126.07 (Ar-C1 Tyr), 116.15 (Ar-C3 and C5 Tyr), 59.08 (Cα Val), 56.19 (Cα Tyr), 55.64 (Cα Phe), 38.86 (CH_2 Phe), 37.91 (CH_2 Tyr), 31.85 (CH Val), 22.40 (CH_3 Ac), 19.56 (CH_3 Val), 18.44 (CH_3 Val).

HRMS (ESI-TOF) *m/z* calculated for $C_{25}H_{31}N_3O_6$ $[M+H]^+$: 470.2271. Found $[M+H]^+$: 470.2271.

M. p.: 98-99 ^{o}C.

Purity (214 nm): 100%, t_r = 11.46 min.

Ac-Tyr-Phe-Val-OCH_3 (2)

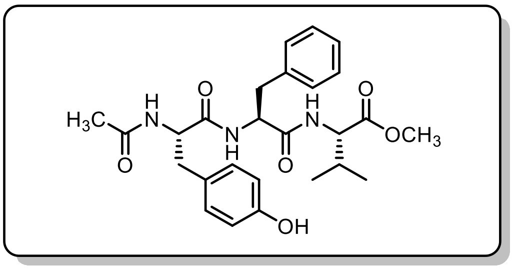

As described in the general peptide synthesis method, starting from 2-chlorotrityl chloride resin (1.55 mmol/g), with HTCU as coupling reagent and using the general cleavage procedure **B**, **2** was obtained as a white solid (45 mg 87%). The final product was purified by semipreparative HPLC (3 mg, 6%).

^{1}H-NMR (500 MHz, CD_3OD): δ 7.33 – 7.15 (m, 5H, ArH Phe), 7.01 (d, *J* = 8.5 Hz, 2H, Ar-H2 and H6 Tyr), 6.68 (d, *J* = 8.5 Hz, 2H, Ar-H3 and H5 Tyr), 4.66 (dd, *J* = 8.3, 6.1 Hz, 1H, Hα Phe), 4.53 (dd, *J* = 8.9, 5.6 Hz, 1H, Hα Tyr), 4.30 (d, J = 6.3 Hz, 1H, Hα Val), 3.70 (s, 3H, $OC\underline{H}_3$) 3.13 (dd, *J* = 13.8, 6.1 Hz, 1H, CH_{2a} Phe), 2.95 – 2.86 (m, 2H, CH_{2a} Tyr + CH_{2b} Phe), 2.66 (dd, *J* = 14.1, 8.9 Hz, 1H, CH_{2b} Tyr), 2.18 – 2.05 (m, 1H, CH Val), 1.88 (s, 3H, $COCH_3$), 0.95 (d, *J* = 3.5 Hz, 3H, CH_3 Val), 0.93 (d, *J* = 3.5 Hz, 3H, CH_3 Val).

^{13}C-NMR (125 MHz, CD_3OD): δ 173.44 (CO), 173.29 (CO), 173.16 (CO), 173.06 (CO), 157.25 (Ar-C4 Tyr), 138.13 (Ar-C1 Phe), 131.18 (Ar-C2 and C6 Tyr), 130.46 (Ar-C3 and C5 Phe), 129.39 (Ar-C2 and C6 Phe), 128.99 (Ar-C1 Tyr), 127.72 (Ar-C4 Phe), 116.17 (Ar-3 and C5 Tyr), 59.31 (Cα Val), 56.22 (Cα Tyr), 55.64 (Cα Phe), 52.47 (OCH_3 Val), 38.90 (CH_2 Phe), 37.90 (CH_2 Tyr), 31.93 (CH Val), 22.39 (CH_3 Ac), 19.42 (CH_3 Val), 18.64 (CH_3 Val).

HRMS (ESI-TOF) *m/z* calculated for $C_{26}H_{34}N_3O_6$ $[M+H]^+$: 484.2442. Found $[M+H]^+$: 484.2447.

M. p.: 75-77 ^oC.

Purity (214 nm): 97.5%, t_r = 12.714 min.

Ac-Ala-Phe-Val-OCH_3 (3)

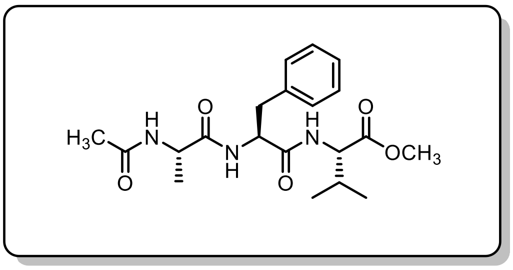

As described in the general peptide synthesis method, starting from 2-chlorotrityl chloride resin (1.55 mmol/g), with HCTU as coupling agent and using the general cleavage procedure **B, 3** was obtained as a white solid (23 mg, 46% crude yield). The product was purified by semipreparative HPLC (5 mg, 10%).

^{1}H-NMR (500 MHz, CD_3OD): δ 7.34 – 7.09 (m, 5H, ArH Phe), 4.68 (dd, *J* = 8.3, 6.1 Hz, 1H, Hα Phe), 4.30 (m, 2H, Hα Val and Ala), 3.70 (s, 3H, OCH_3) 3.16 (dd, *J* = 13.9, 6.1 Hz, 1H, CH_2 Phe), 2.96 (dd, *J* = 13.9, 8.3 Hz, 1H, CH_2 Phe), 2.13 (dd, *J* = 13.3, 6.8 Hz, 1H, CH Val), 1.94 (s, 3H, CH_3 Ac), 1.25 (d, *J* = 7.2 Hz, 3H, CH_3 Ala), 0.94 (dd, *J* = 6.8, 3.5 Hz, 6H, 2xCH_3 Val).

^{13}C-NMR (125 MHz, CD_3OD): δ 174.84 (CO) , 173.45 (CO), 173.17 (CO), 172.86 (CO), 138.17 (Ar-C4 Phe), 130.43 (Ar-C3 and C5 Phe), 129.39 (Ar-C2 and C6 Phe), 127.72 (Ar-C1 Phe), 59.33 (Cα Val), 55.58 (Cα Phe), 52.46 (Cα Ala), 50.44 (OCH_3), 38.61 (CH_2 Tyr), 31.90 (CH Val), 22.38 (CH_3 Ac), 19.41 (CH_3 Val), 18.59 (CH_3 Val), 17.72 (CH_3 Ala).

HRMS (ESI-TOF) *m/z* calculated for $C_{20}H_{30}N_3O_5$ $[M+H]^+$: 392.2186. Found $[M+H]^+$: 392.2172.

M. p.: 86-88 ^oC.

Purity (214 nm): 100 %, t_r = 11.961 min.

Ac-Tyr-Ala-Val-OCH₃ (4)

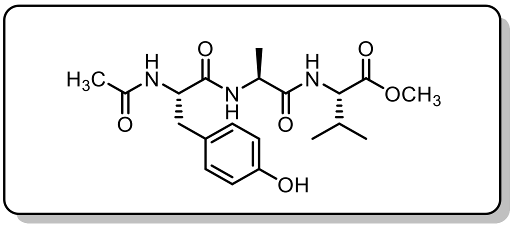

As described in the general peptide synthesis method, starting from 2-chlorotrityl chloride resin (1.55 mmol/g), with HCTU as coupling agent and using the general cleavage procedure **B**, **4** was obtained as a white solid (31 mg, 77% crude yield). The product was purified by semi-preparative HPLC (2.5 mg, 6%).

^{1}H-NMR (500 MHz, CD_3OD): δ 7.07 (d, *J* = 8.6 Hz, 2H, Ar-H2 and H6 Tyr), 6.70 (d, *J* = 8.6 Hz, 2H, Ar-H3 and H5 Tyr), 4.56 (dd, *J* = 9.2, 5.3 Hz, 1H, Hα Tyr), 4.43 (q, *J* = 7.1 Hz, 1H, Hα Ala), 4.12 (ddd, *J* = 17.7, 11.4, 5.3 Hz, 1H, Hα Val), 3.73 (s, 3H, OCH_3), 3.05 (dd, *J* = 14.1, 5.3 Hz, 1H, CH_2 Tyr), 2.78 (dd, *J* = 14.1, 9.2 Hz, 1H, CH_2 Tyr), 2.16 (qd, *J* = 12.9, 6.9 Hz, 1H, CH Val), 1.91 (s, 3H, CH_3 Ac),1.36 (d, *J* = 7.1 Hz, 3H, CH_3 Ala), 1.01 – 0.96 (m, 6H, 2xCH_3 Val).

^{13}C-NMR (125 MHz, CD_3OD): δ 174.91 (CO), 173.60 (CO), 173.43 (CO), 173.20 (CO), 157.28 (Ar-C4 Tyr), 131.25 (Ar-C4 and C6 Tyr), 129.03 (Ar-C1 Tyr), 116.16 (Ar-C3 and C5 Tyr), 59.24 (Cα Val), 56.22 (Cα Tyr), 52.46 (Cα Ala), 50.14 (OCH_3), 38.08 (CH_2 Tyr), 30.75 (CH Val), 22.37 (CH_3 Ac), 19.47 (CH_3 Val), 18.55 (CH_3 Val), 18.05 (CH_3 Ala).

HRMS (ESI-TOF) *m/z* calculated for $C_{20}H_{30}N_3O_6$ $[M+H]^+$: 408.2129. Found $[M+H]^+$: 408.2128.

M. p.: 84-86 ^{o}C.

Purity (214 nm): 100 %, t_r = 9.599 min.

Ac-Tyr-Phe-Ala-OCH_3 (5)

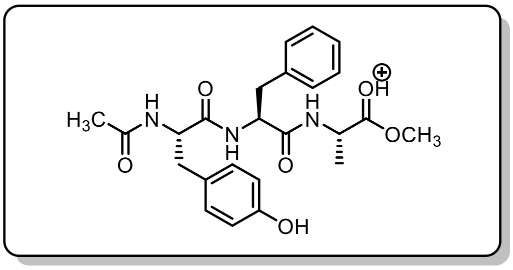

As described in the general peptide synthesis method, starting from 2-chlorotrityl chloride resin (1.55 mmol/g), with HCTU as coupling agent and using the general cleavage procedure **B**, **5** was obtained as a white solid (33 mg, 82%, crude yield). The product was purified by semipreparative HPLC (2.5 mg, 6%).

^{1}H-NMR (500 MHz, CD_3OD): δ 7.34 – 7.16 (m, 5H, ArH Phe), 7.00 (d, J = 8.6 Hz, 2H, Ar-H2 an H6 Tyr), 6.68 (d, J = 8.6 Hz, 2H, Ar-H3 and H5 Tyr), 4.62 (dd, J = 8.5, 5.6 Hz, 1H, Hα Phe), 4.49 (dd, J = 8.8, 6.0 Hz, 1H, Hα Tyr), 4.39 (d, J = 7.3 Hz, 1H, Hα Ala), 3.71 (s, 3H, OCH_3), 3.17 (dd, J = 13.9, 5.6 Hz, 1H, CH_{2a} Phe), 2.97 – 2.86 (m, 2H, CH_{2b} Phe and CH_{2a} Tyr), 2.71 (dd, J = 14.0, 8.8 Hz, 1H, CH_{2b} Tyr), 1.88 (s, 3H, CH_3 Ac), 1.39 (d, J = 7.3 Hz, 3H, CH_3 Ala).

^{13}C-NMR (125 MHz, CD_3OD): δ 174.30 (CO), 173.43 (CO), 173.15 (CO), 172.86 (CO), 157.28 (Ar-C4 Tyr), 138.23 (Ar-C1 Phe), 131.17 (Ar-C2 and C6 Tyr), 130.50 (Ar-C3 and C5 Phe), 129.38 (Ar-C2 and C6 Phe), 128.98 (Ar-C1 Tyr) , 127.70 (Ar-C4 Phe), 116.17 (Ar-C3 and C5 Tyr), 56.42 (Cα Tyr), 55.48 (Cα Phe), 52.75 (Cα Ala), 49.49 (OCH_3 overlapped with CH_3OH), 39.15 (CH_2 Phe), 38.13 (CH_2 Tyr), 22.39 (CH_3 Ac), 17.42 (CH_3 Ala).

MS (ESI-TOF) *m/z* calculated for $C_{24}H_{30}N_3O_6$ $[M+H]^+$: 456.21. Found $[M+H]^+$: 456.20

M. p.: 83-85 ^{o}C.

Purity (214 nm): 100 %, t_r = 10.742 min.

Ac-Tyr-Phe-Ser-OCH_3 (6)

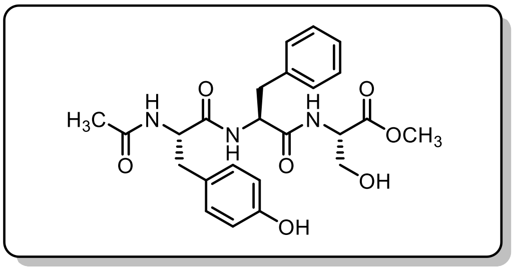

As described in the general peptide synthesis method, starting from 2-chlorotrityl chloride resin (1.33 mmol/g), with HCTU as coupling agent and using the general cleavage procedure **B, 6** was obtained as a white solid (31 mg, 68%, crude yield). The product was purified by semipreparative HPLC (0.9 mg, 2%).

HRMS (ESI-TOF) *m/z* calculated for $C_{24}H_{29}N_3O_7$ $[M+H]^+$: 472.2078. Found $[M+H]^+$: 472.2082.

Purity (214 nm): 100 %, t_r = 9.74 min.

Ac-Tyr-2-2NaI-Val-OCH$_3$ (7)

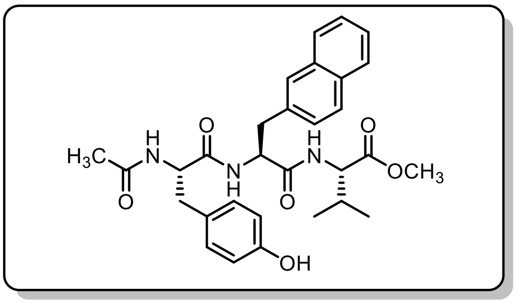

As described in the general peptide synthesis method, starting from 2-chlorotrityl chloride resin (1.55 mmol/g), with HATU as coupling agent and using the general cleavage procedure **B**, (repeating Fmoc-2NaI-OH and Fmoc-Tyr-OH coupling after the first cycle), **7** was obtained as a white solid (23 mg, 46%, crude yield). The product was purified by semipreparative HPLC (4 mg, 6%).

^{1}H-NMR (500 MHz, CD$_3$OD): δ 7.82 – 7.77 (m, 3H, ArH 2NaI), 7.68 (s, 1H, Ar-H1 2NaI), 7.48 – 7.41 (m, 2H, ArH 2NaI), 7.39 (dd, *J* = 8.4, 1.8 Hz, 1H, ArH 2NaI), 6.98 (d, *J* = 8.6 Hz, 2H, Ar-2 and H6 Tyr), 6.66 (d, *J* = 8.6 Hz, 2H, Ar-H3 and H5), 4.81 (dd, *J* = 11.6, 9.9 Hz, 1H, Hα 2NaI), 4.53 (dd, *J* = 8.8, 5.7 Hz, 1H, Hα Tyr), 4.29 (d, *J* = 6.3 Hz, 1H, Hα Val), 3.51 (s, 3H, OCH$_3$), 3.28 (dd, *J* = 13.8, 6.4 Hz, 1H, CH$_{2a}$ 2NaI), 3.10 (dd, *J* = 13.8, 8.1 Hz, 1H, CH$_{2b}$ 2NaI), 2.94 (dd, *J* = 14.1, 5.7 Hz, 1H, CH$_{2a}$ Tyr), 2.69 (dd, *J* = 14.1, 8.8 Hz, 1H, CH$_{2b}$ Tyr), 2.09 (d, *J* = 6.6 Hz, 1H, CH Val), 1.77 (s, 3H, CH$_3$ Ac), 0.96 – 0.90 (m, 6H, 2xCH$_3$ Val).

^{13}C-NMR (125 MHz, CD$_3$OD): δ 173.41 (CO), 173.35 (CO), 173.15 (CO), 173.06 (CO), 157.23 (Ar-C4 Tyr), 137.29 (ArC 2NaI), 134.96 (ArC 2NaI), 131.18 (Ar-C5 and C8 2NaI), 130.34 (Ar-C6 and C7 2NaI), 130.02 (Ar-C2 and C6 Tyr), 128.99 (Ar-C1 2NaI), 128.01 (Ar-C1 Tyr), 116.16 (Ar-C3 and C5 Tyr), 59.30 (Cα Val), 56.24 (Cα 2NaI), 55.71 (Cα Tyr), 52.45 (OCH$_3$), 38.49 (CH$_2$ 2NaI), 37.88 (CH$_2$ Tyr), 31.93 (CH Val), 22.38 (CH$_3$ Ac), 19.42 (CH$_3$ Val), 18.64 (CH$_3$ Val). (Three quaternary carbons were not observed).

HRMS (ESI-TOF) *m/z* calculated for $C_{30}H_{36}N_3O_6$ $[M+H]^+$: 534.2599. Found $[M+H]^+$: 534.2608.

M. p.: 90-92 ^{o}C.

Purity (214 nm): 100 %, t_r = 14.595 min.

Ac-Tyr-2NaI-Ser-OCH₃ (8)

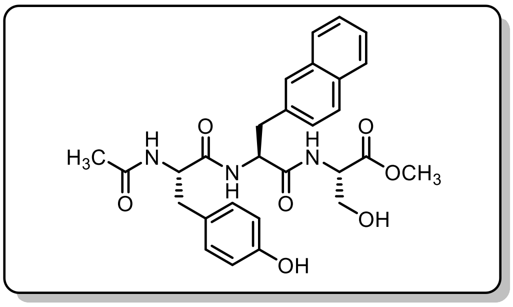

As described in the general peptide synthesis method, starting from 2-chlorotrityl chloride resin (1.55 mmol/g), with HATU as coupling agent and using the general cleavage procedure **B**, **8** was obtained as a white solid (31 mg, 62%, crude yield). The product was purified by semipreparative HPLC (1.1 mg, 2%).

^{1}H-NMR (500 MHz, CD_3OD): δ 7.83 – 7.79 (m, 3H, Ar-H4, H5 and H8 2NaI), 7.71 (d, *J* = 0.6 Hz, 1H, Ar-H1 2NaI), 7.48 – 7.40 (m, 3H, ArH 2NaI), 6.94 (d, *J* = 8.6 Hz, 2H, Ar-H2 and H6 Tyr), 6.64 (d, *J* = 8.6 Hz, 2H, Ar-H3 and H5 Tyr), 4.79 (dd, *J* = 12.0, 4.5 Hz, 1H, $H_α$ 2NaI), 4.46 (a t, *J* = 4.5 Hz, 1H, $H_α$ Ser), 4.46 (dd, *J* = 9.1, 5.7 Hz, 1H, Hα Tyr), 3.90 (dd, *J* = 11.4, 4.8 Hz, 1H, CH_{2a} Ser), 3.81 (dd, *J* = 11.4, 4.2 Hz, 1H, CH_{2b} Ser), 3.64 (s, 3H, OCH_3), 3.37 (dd, *J* = 13.9, 5.7 Hz, 1H, CH_2 2NaI, partially overlapped with H_2O), 3.12 (dd, *J* = 13.9, 8.7 Hz, 1H, CH_{2b} 2NaI), 2.88 (dd, *J* = 14.1, 5.7 Hz, 1H, CH_{2a} Tyr), 2.67 (dd, *J* = 14.1, 9.1 Hz, 1H, CH_{2b} Tyr) 1.77 (s, 3H, CH_3 Ac).

^{13}C-NMR (125 MHz, CD_3OD): δ 173.64 (CO), 173.15 (CO), 171.89 (CO), 170.77 (CO), 157.21 (Ar-C4 Tyr), 136.78 (ArC 2NaI), 133.95 (ArC 2NaI), 131.09 (Ar-C2 and C6 Tyr), 129.21 (Ar-C1 Tyr), 129.10 (ArC 2NaI), 129.02 (ArC 2NaI), 128.95 (ArC 2NaI), 128.75 (ArC 2NaI), 128.68 (ArC 2NaI), 128.59 (ArC 2NaI), 126.98 (ArC 2NaI), 126.59 (ArC 2NaI), 116.15 (Ar-C3 and C5 Tyr), 62.86 (CH_2OH Ser), 56.53 (CHα Ser), 56.26 (CHα Tyr), 55.66 (CHα 2NaI), 52.75 (OCH_3) , 38.93 (CH_2 Phe), 37.77 (CH_2 Tyr), 22.25 (CH_3 Ac).

HRMS (ESI-TOF) *m/z* calculated for $C_{28}H_{32}N_3O_7$ $[M+H]^+$: 522.2235. Found $[M+H]^+$: 522.2241.

Purity (214 nm): 94 %, t_r = 12.426 min.

1. NMR spectra experiments for representative key compounds 2 and 3

(2) Ac-Tyr-Phe-Val-OCH$_3$

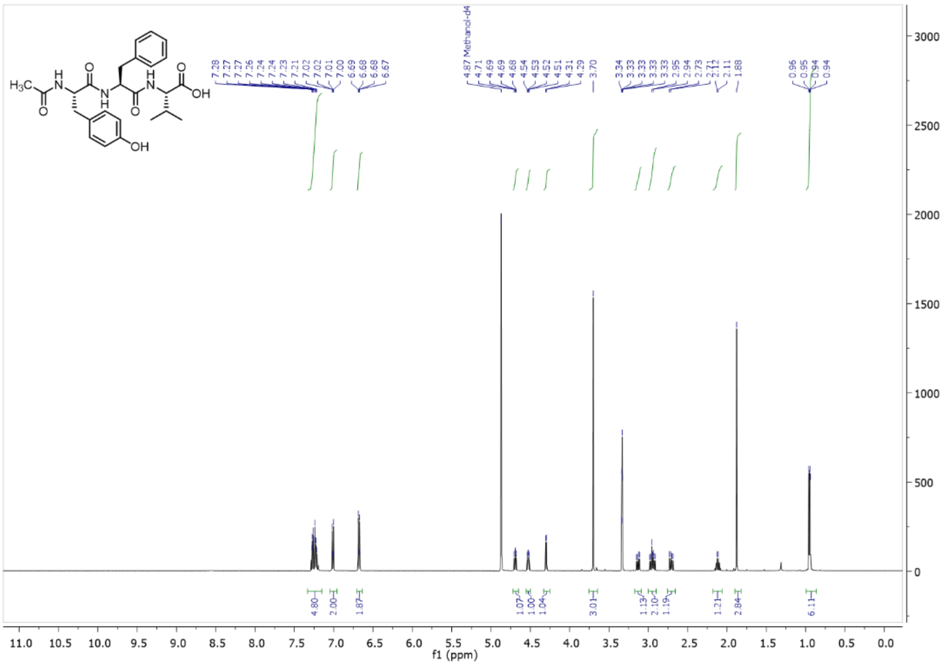

Figure S 7. *^{1}H-NMR spectra for compound **2***

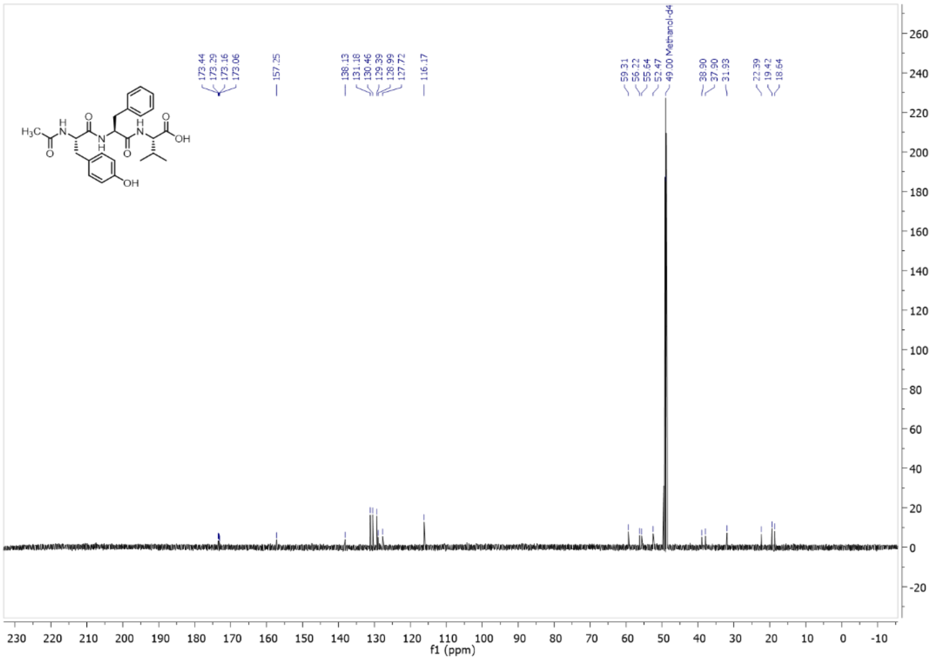

Figure S 8. *^{13}C-NMR spectra for compound **2***

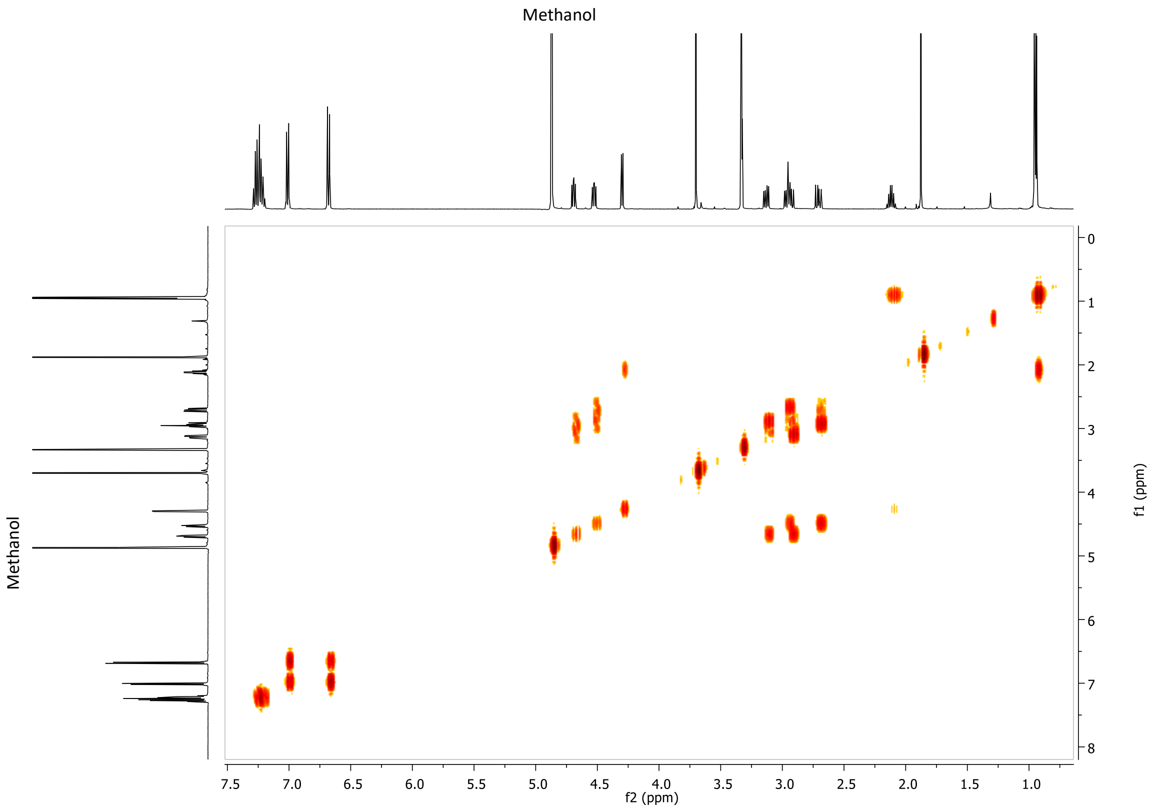

Figure S 9. *COSY spectra for compound **2***

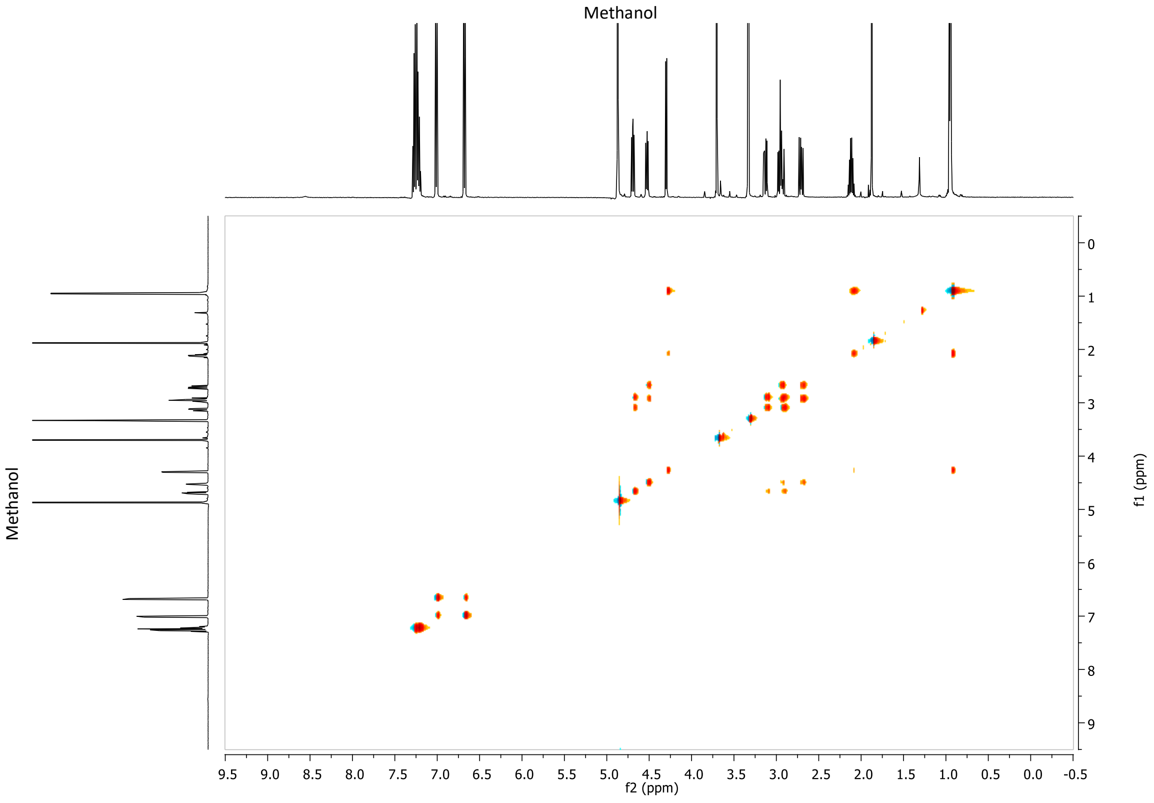

Figure S 10. *TOCSY spectra for compound **2***

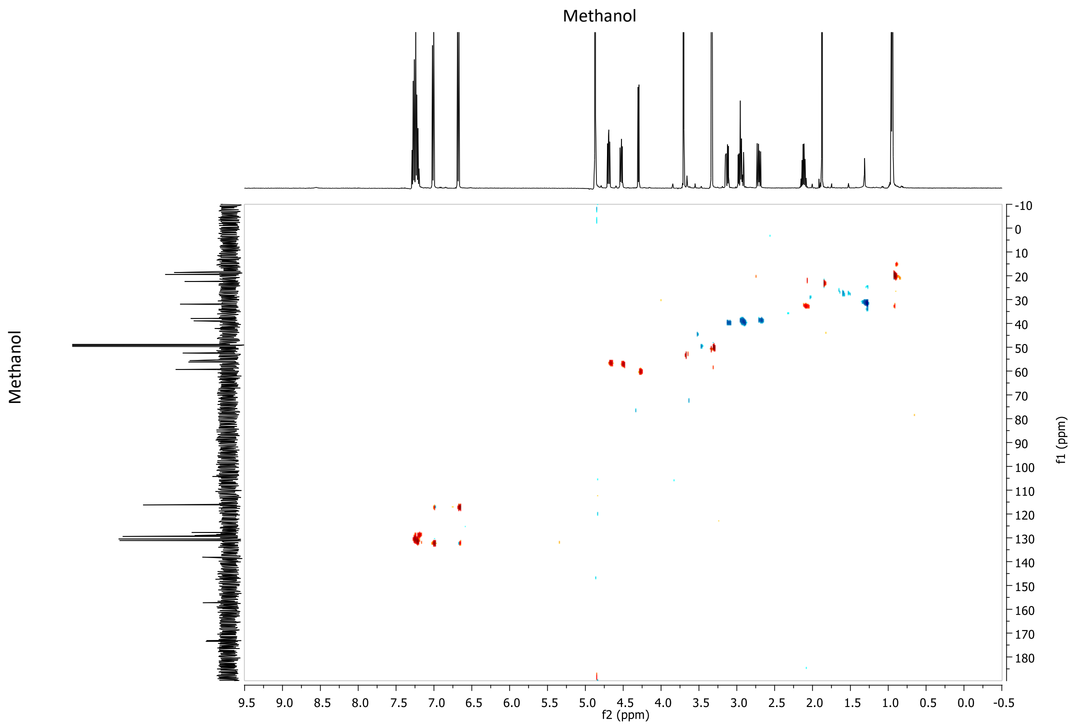

Figure S 11. *gHSQC spectra for compound* ***2***

(3) Ac-Ala-Phe-Val-OCH$_3$

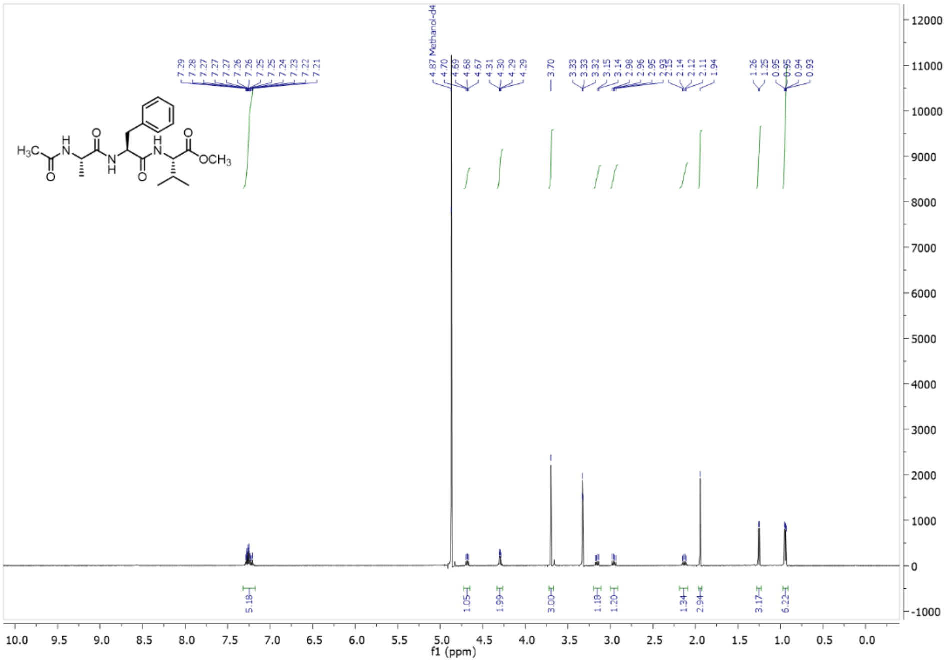

Figure S 12*.* *^{1}H-NMR spectra for compound* ***3***

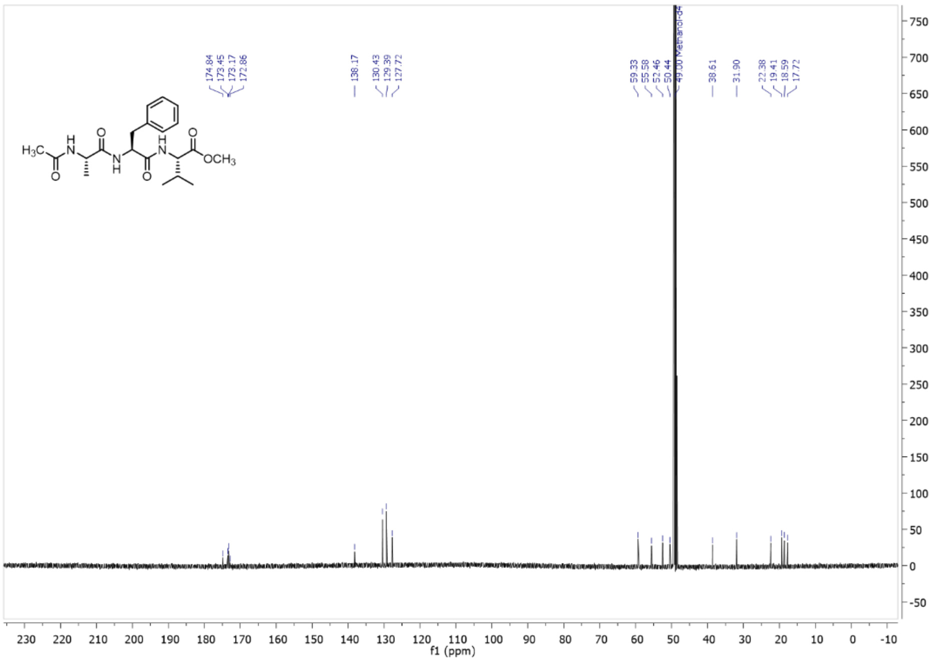

Figure S 13. *¹H-NMR spectra for compound **3***

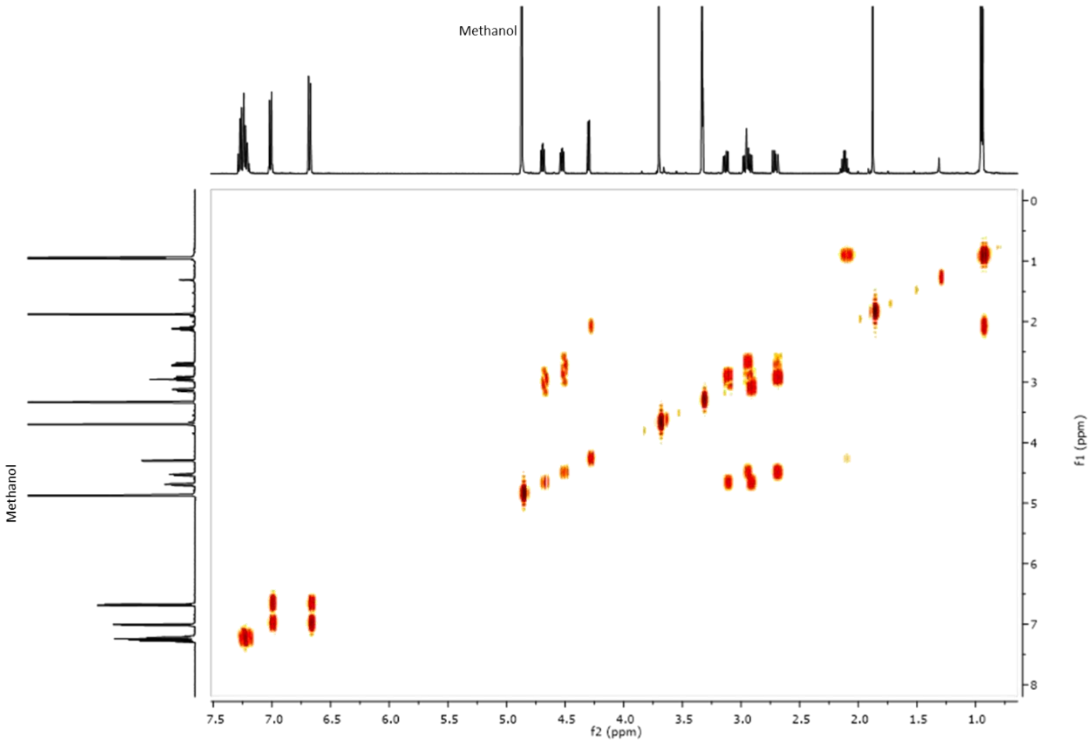

Figure S 14. *COSY spectra for compound **3***

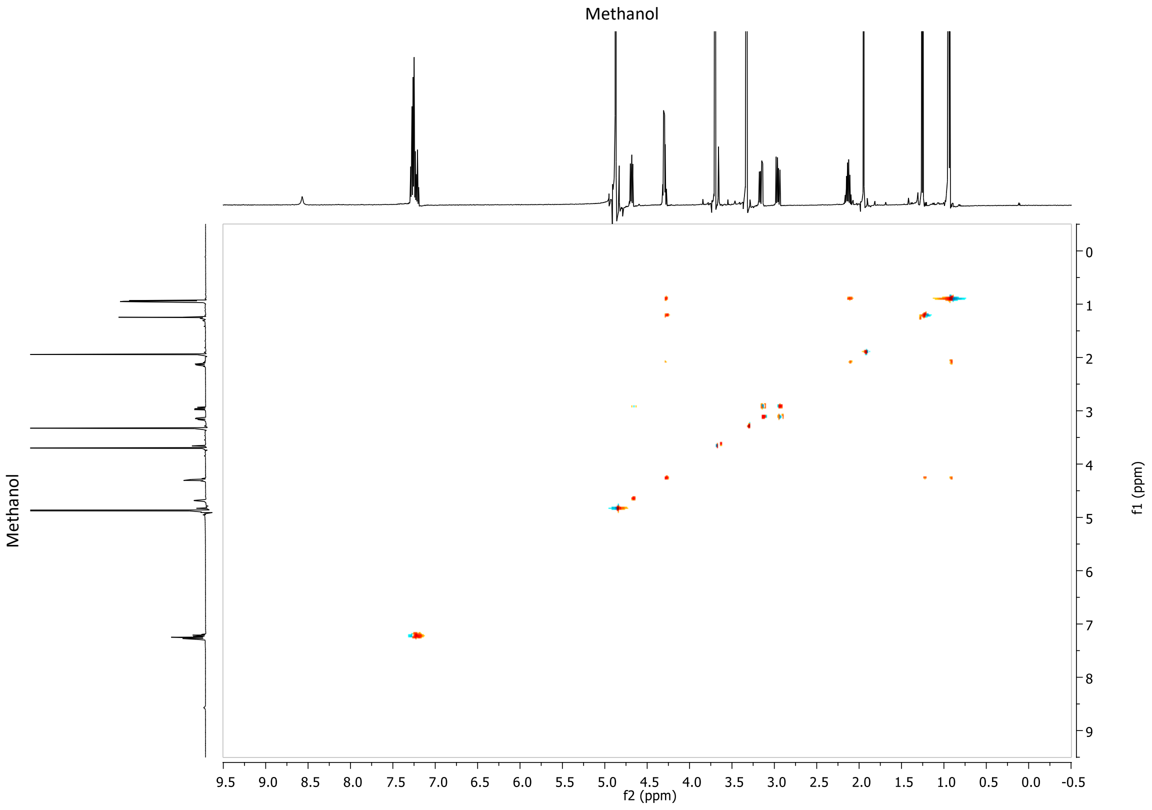

Figure S 15. *TOCSY spectra for compound **3***

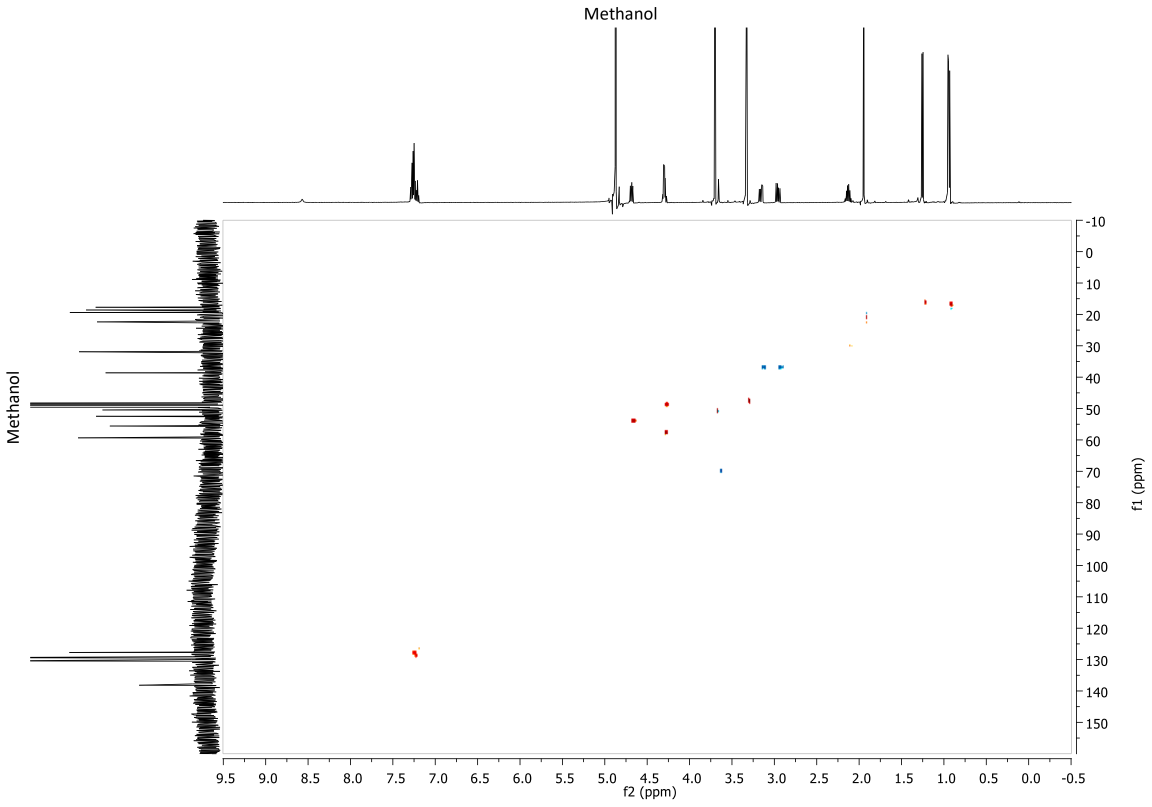

Figure S 16. *gHSQC spectra for compound **3***

6 Bibliography

1. Kozakov, D.; Grove, L. E.; Hall, D. R.; Bohnuud, T.; Mottarella, S. E.; Luo, L.; Xia, B.; Beglov, D.; Vajda, S. The FTMap family of web servers for determining and characterizing ligand-binding hot spots of proteins. *Nature Protocols.* **2015**, *10*, 733-755.

2. Tuncbag, N.; Keskin, O.; Gursoy, A. HotPoint: hot spot prediction server for protein interfaces. *Nucleic Acids Res.* **2010**, *38*, 402.

3. Tuncbag, N.; Gursoy, A.; Keskin, O. Identification of computational hot spots in protein interfaces: combining solvent accessibility and inter-residue potentials improves the accuracy. *Bioinformatics.* **2009**, *25*, 1513-1520.

4. Jo, S.; Kim, T.; Iyer, V. G.; Im, W. CHARMM-GUI: A web-based graphical user interface for CHARMM. *J. Comput. Chem.* **2008**, *29*, 1859-1865.

5. Huang, J.; Rauscher, S.; Nawrocki, G.; Ran, T.; Feig, M.; de Groot, B.,L.; Grubmüller, H.; MacKerell, A. D. CHARMM36m: an improved force field for folded and intrinsically disordered proteins. *Nat. Methods.* **2017**, *14*, 71-73.

6. Phillips, J. C.; Braun, R.; Wang, W.; Gumbart, J.; Tajkhorshid, E.; Villa, E.; Chipot, C.; Skeel, R. D.; Kalé, L.; Schulten, K. Scalable molecular dynamics with NAMD. *J. Comput. Chem.* **2005**, *26*, 1781-1802.

7. Troyano-Suárez, N.; del Nogal-Avila, M.; Mora, I.; Sosa, P.; López-Ongil, S.; Rodriguez-Puyol, D.; Olmos, G.; Ruíz-Torres, M. P. Glucose Oxidase Induces Cellular Senescence in Immortal Renal Cells through ILK by Downregulating Klotho Gene Expression. *Oxid Med. Cell. Longev.* **2015**, *2015*, 416738.

8. Cano-Peñalver, J. L.; Griera, M.; García-Jerez, A.; Hatem-Vaquero, M.; Ruiz-Torres, M. P.; Rodríguez-Puyol, D.; Frutos, S.; Rodríguez-Puyol, M. Renal Integrin-Linked Kinase Depletion Induces Kidney cGMP-Axis Upregulation: Consequences on Basal and Acutely Damaged Renal Function. *Mol. Med.* **2016**, *21*, 873-885.

9. Troyano, N.; Nogal, M. D.; Mora, I.; Diaz-Naves, M.; Lopez-Carrillo, N.; Sosa, P.; Rodriguez-Puyol, D.; Olmos, G.; Ruiz-Torres, M. P. Hyperphosphatemia induces cellular senescence in human aorta smooth muscle cells through integrin linked kinase (ILK) up-regulation. *Mech. Ageing Dev.* **2015**, *152*, 43-55.

10. de Frutos, S.; Luengo, A.; Garcia-Jerez, A.; Hatem-Vaquero, M.; Griera, M.; O'Valle, F.; Rodriguez-Puyol, M.; Rodriguez-Puyol, D.; Calleros, L. Chronic kidney disease induced by an adenine rich diet upregulates integrin linked kinase (ILK) and its depletion prevents the disease progression. *Biochim. Biophys. Acta Mol. Basis Dis.* **2019**, *1865*, 1284-1297.

11. Mamuya, F. A.; Cano-Peñalver, J. L.; Li, W.; Rodriguez Puyol, D.; Rodriguez Puyol, M.; Brown, D.; de Frutos, S.; Lu, H. A. ILK and cytoskeletal architecture: an important determinant of AQP2 recycling and subsequent entry into the exocytotic pathway. *Am. J. Physiol. Renal Physiol.* **2016**, *311*, F1346-F1357.
